# Magnesium Supplementation and Blood Pressure: A Systematic Review and Meta-Analysis of Randomized Controlled Trials

**DOI:** 10.1161/HYPERTENSIONAHA.125.25129

**Published:** 2025-09-26

**Authors:** Zoe Argeros, Xiaoye Xu, Buna Bhandari, Katie Harris, Rhian M. Touyz, Aletta E. Schutte

**Affiliations:** School of Population Health, University of New South Wales, Sydney, Australia (Z.A., X.X., A.E.S.).; The George Institute for Global Health, Sydney, Australia (X.X., K.H., A.E.S.).; Department of Global Health and Population, Harvard T H Chan School of Public Health, Boston (B.B.).; Florida State University College of Nursing, Tallahassee (B.B.).; Department of medicine and Department of family medicine, Research Institute of McGill University Health Centre, McGill University, Montreal, Canada (R.M.T.).

**Keywords:** blood pressure, cardiovascular disease, diet, hypertension, magnesium

## Abstract

**BACKGROUND:**

There are inconsistent reports regarding the effect of magnesium intake on blood pressure (BP) across hypertensive and normotensive populations.

**METHODS:**

We performed a meta-analysis and dose-response analysis to explore the relationship between magnesium supplementation and BP in randomized-controlled trials with a duration of ≥4 weeks, using a cubic spline regression model.

**RESULTS:**

Thirty-eight randomized controlled trials involving 2709 participants were eligible for inclusion. Studies included an elemental magnesium dose from 82.3 mg to 637 mg with a median dose of 365 mg and a median intervention period of 12 weeks. Mean differences of changes in BP were calculated by random effects meta-analysis. Magnesium intake resulted in a reduction in systolic BP of −2.81 mm Hg (95% CI, −4.32 to −1.29) and diastolic BP by −2.05 mm Hg (95% CI, −3.23 to −0.88) compared with placebo. Hypertensive individuals on BP-lowering medication and individuals with hypomagnesemia yielded greater systolic BP reductions of −7.68 and −5.97 mm Hg, respectively (*P*<0.05), and diastolic BP reductions of −2.96 and −4.75 mm Hg, respectively (*P*<0.05). In normotensive groups, statistical significance was not reached. We identified high heterogeneity across studies. We found no dose-response relationship between magnesium and BP changes (all *P*≥0.20).

**CONCLUSIONS:**

Our findings support the beneficial effect of magnesium on reducing BP among populations with hypertension and hypomagnesemia, although effects should be interpreted with caution due to high heterogeneity of studies. Larger, well-designed studies assessing higher magnesium doses are needed to refine the dose-response relationship between magnesium intake and BP and identify potential optimal supplementation strategies for subpopulations.

**NOVELTY AND RELEVANCE**

**What Is New?**

This extensive meta-analysis examines magnesium supplementation and blood pressure, including 38 randomized controlled trials and 2709 participants. It evaluates subgroups by hypertension status, medication use, and serum magnesium levels.

**What Is Relevant?**

Magnesium significantly lowers blood pressure in individuals with hypertension and hypomagnesemia, especially those on antihypertensive medication. No significant effect was found in normotensive individuals.

**Clinical/Pathophysiological Implications?**

Magnesium may enhance blood pressure control through vascular and renal mechanisms. These findings support targeted supplementation strategies and highlight the need for magnesium status assessment in hypertension management.

Raised blood pressure (BP) is the leading risk factor for cardiovascular disease and preventable deaths, contributing to 19.2% of all deaths in 2019.^1^ Lifestyle modification, including dietary interventions, is important for preventing raised BP and acting as the first line of treatment for hypertension.^2^

Consistent evidence for dietary interventions to lower BP and prevent hypertension mostly focuses on decreasing sodium intake and increasing potassium intake.^3^ The Dietary Approaches to Stop Hypertension diet centers on a more holistic approach through food intake that would increase potassium, calcium, and magnesium while lowering sodium.^4,5^ Despite the consistent evidence on the importance of sodium^6,7^ and potassium,^8^ the evidence on the impact of magnesium alone on reducing BP has been inconsistent.^9^

Magnesium may lower BP through several mechanisms. These include regulating cardiac rhythm,^10^ reducing vascular tone,^10^ inhibiting the renin angiotensin aldosterone system,^11^ increasing nitric oxide release and endothelial prostaglandin I_2_ secretion,^12^ decreasing reabsorption of sodium^11^ and enhancing the effect of BP-lowering medication.^13,14^ The potential benefits of magnesium on BP may also be greater among individuals with hypomagnesemia.^15^

Despite these potential benefits, the latest evidence review by the United States Food and Drug Association in 2022 identified supportive yet inconclusive evidence that diets with adequate magnesium reduce hypertension.^9^ Of the meta-analyses conducted to date, 2 of those reviewing randomized controlled trials found small yet significant reductions in systolic BP (SBP) and diastolic BP (DBP),^16,17^ 1 found magnesium supplementation reduced DBP but not SBP among hypertensive individuals,^18^ and 1 review found no effect of oral magnesium supplementation on BP.^17^

The latest meta-analysis conducted in 2017 by Dibaba et al^13^ assessed the effect of magnesium supplementation on BP in those with underlying preclinical conditions (insulin resistance, prediabetes, and noncommunicable chronic disease). Eleven randomized controlled trials, with a total of 543 participants and a mean follow-up of 3.6 months, were included, which found that magnesium supplementation reduced SBP by −4.18 mm Hg and DBP by −2.27 mm Hg compared with control groups.

Although there are promising findings for magnesium on BP-lowering, a comprehensive meta-analysis assessing the effects of magnesium intake on individuals based on hypertension and medication status, along with assessing the dose-response relationship between magnesium intake and BP in both normotensive and hypertensive individuals, has not been undertaken. We, therefore, conducted a meta-analysis and dose-response meta-analysis of randomized controlled trials to quantifiably analyze the association between magnesium supplementation and BP among adults (with and without clinical conditions) with BP in the normotensive and hypertensive BP ranges. We did not include studies that evaluated dietary magnesium intake on BP.

## Methods

### Data Availability

We conducted this systematic review aligning with the Preferred Reporting Items for Systematic Reviews and Meta-Analyses (PRISMA) guidelines, after registering it in the International Prospective Register of Systematic Reviews (No. CRD42024569285). All supporting data are available within the main text and Supplemental Material.

### Data Sources and Search

We conducted a systematic search of online databases (Medline, EMBASE, Web of Science, Scopus, and Cumulative Index to Nursing and Allied Health Literature) for articles published on or before May 19, 2024, with English restriction. We identified randomized controlled trials and observational studies that reported on the effect of magnesium intake on SBP and DBP among humans using the key words magnesium, BP, hypertension, diet, intake, supplement, clinical trials, randomized controlled trials, cohort, case-control to identify potentially relevant studies. Citations in past systematic reviews were also analyzed to identify additional studies. Additional details of our search strategy are reported in Table S1.

### Screening

Search results from all databases were imported into the web-based software Covidence (Covidence systematic review software, Veritas Health Innovation, Melbourne, Australia), and duplicates were automatically removed.

**Figure 1.**
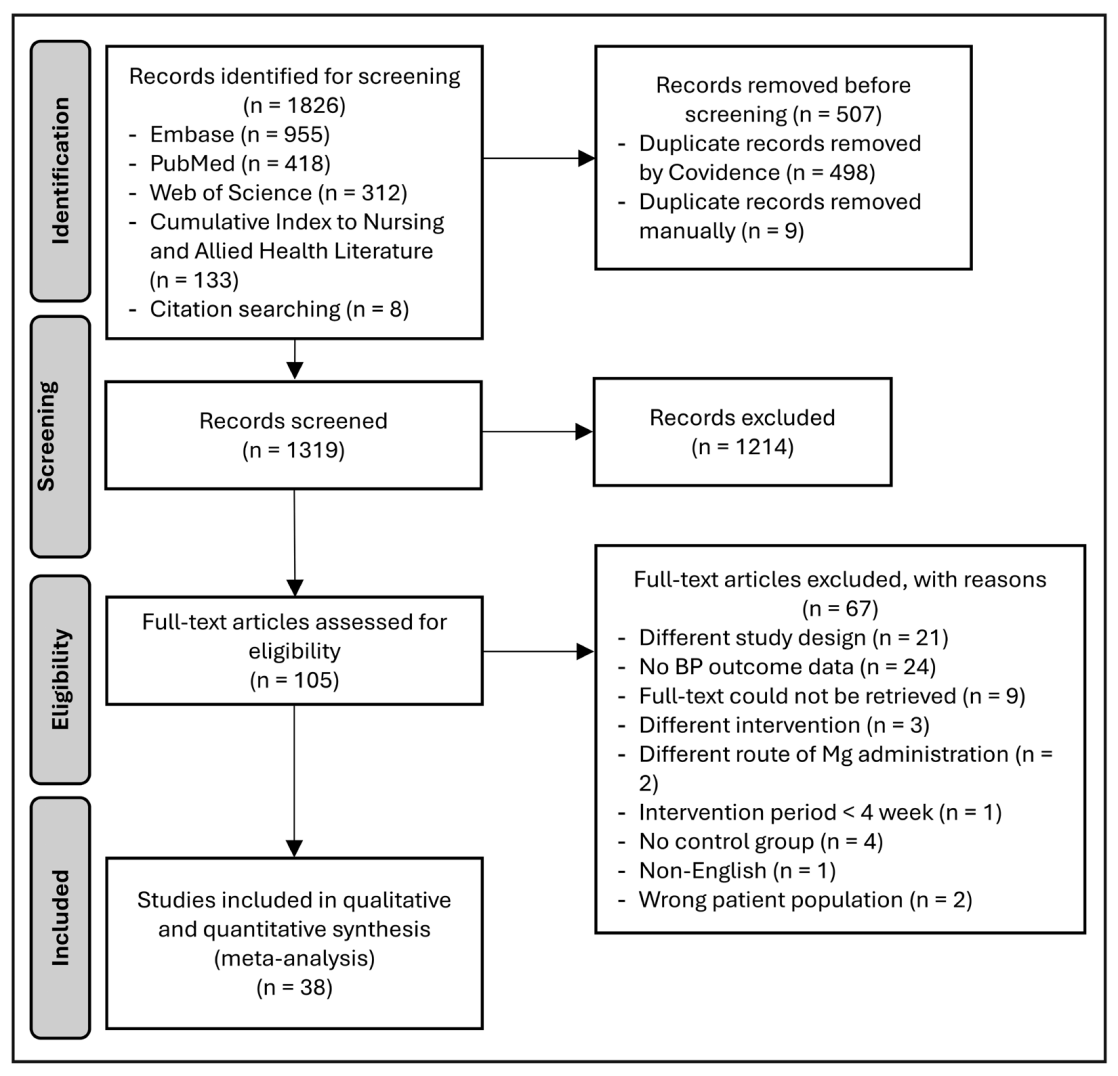
**PRISMA flow diagram of search results and eligible articles.** BP indicates blood pressure.

Two authors (Z.A. and B.B.) independently screened all study titles and abstracts against inclusion and exclusion criteria, with conflicts resolved by a third author (X.X.). Full-text articles were also reviewed using the same process to determine final studies for inclusion. Details, including reasons for the exclusion of the study, are presented in the PRISMA flow diagram in Figure 1.

### Study Selection

We used the Population, Intervention, Comparator and Outcome framework to determine the eligibility criteria of our study. A study was eligible for this systematic review and meta-analysis if it (1) was based on a randomized controlled trial, cohort, case-control or nested case-control study; (2) included a population of adults aged 18 years and above; (3) evaluated the relationship between magnesium intake and SBP and DBP during follow up; (4) included an intervention period of at least 4 weeks^16^; and (5) included the presence of a placebo or control group.

We excluded studies that used a cross-sectional design, that enrolled participants who were pregnant, where medication or dietary intervention was altered throughout the intervention period, and studies that did not report measurements of magnesium intake and pre- and post-SBP and DBP readings.

### Data Extraction

One author (Z.A.) extracted data for all selected studies, and this was reviewed by 2 other authors (A.E.S. and B.B.). The extracted data included the author names, year of publication, country, study design (crossover or parallel), number of participants in each study arm, sex of participants, age of participants, comorbidity characteristics, hypertension and BP lowering medication status, intervention duration, type and quantity of magnesium treatment, type of placebo, position of BP measurement (seated, supine, standing or 24-hour ambulatory), baseline and end of study mean±SD SBP and DBP for intervention and control arms, and mean change±SD in SBP and DBP per study arm where available.

Magnesium dose was converted and reported as milligrams of elemental magnesium to assist meta-analysis and dose-response analysis. When the SD of SBP and DBP was not directly reported, it was calculated from the SEM according to the Cochrane Handbook for Systematic Reviews and Interventions.^19^ Where mean change±SD in SBP and DBP was not reported and SD for the mean change was unavailable, SD of mean change was calculated using methods outlined by Fu et al^20^ with the correlation between BP at baseline and end of study assumed as *r*=0.5 as per Dibaba et al.^13^

### Risk of Bias Assessment

An assessment of study quality was conducted using the Revised Cochrane risk-of-bias tool for randomized trials and the Revised Cochrane risk-of-bias tool for randomized crossover trials.^21^ Bias was assessed based on 5 domains, including bias during randomization, deviations from the intended interventions, incomplete outcome data, outcome measurement, and selective reporting. For crossover studies, bias arising from period and carryover effects was also evaluated. Each domain was characterized as having low risk of bias, some concerns, or a high risk of bias. A study was assigned an overall low risk of bias if all domains were low risk, moderate risk if up to 3 domains were of some concern, and high risk when at least 1 domain was high risk or when 4 or more domains were of some concern.

### Data Synthesis and Statistical Analysis

All the included studies were randomized controlled trials that assessed the effect of magnesium intake on BP in individuals. We did not find a cohort, case-control, or nested case-control study that met the inclusion criteria.

To analyze the overall effects of magnesium on BP, we compared the mean changes of SBP and DBP between intervention and placebo groups by conducting a meta-analysis to pool weighted mean differences and 95% CIs using an inverse-variance weighted random-effects model. Between-study heterogeneity was determined by calculating the *I*^*2*^ statistic, where ≤40% may indicate low heterogeneity, 30% to 60% may represent moderate heterogeneity, 50% to 90% may indicate substantial heterogeneity, and 75% to 100% indicates considerable heterogeneity.^19^

To explore heterogeneity between studies and assess the robustness of the meta-analysis, we conducted subgroup analyses including by hypertension and medication status (normotensive, hypertensive, hypertensive with medication, hypertensive with no medication), study design (crossover or parallel), modality of BP measurement (seated, supine, standing, 24-hour ambulatory), and serum magnesium status (hypomagnesemia or normomagnesemia). Multiple adjusted mixed-effects meta-regression was further used to test heterogeneity by examining whether the proportion of male participants, mean age of participants, magnesium supplementation dose, intervention duration, and baseline SBP were associated with SBP and DBP effect sizes. Publication bias was assessed through visual inspection of the funnel plots and the Egger regression test.

We assessed the dose-response relationship of magnesium dose with SBP and DBP change using restricted cubic splines of magnesium with 3 knots at fixed percentiles (10%, 50%, and 90%) with no a priori assumptions made about the shape of the association. Separate splines were fit to assess the dose-response relationship for the intervention and control arms of the trials. The dose-response relationship was tested for both linearity and nonlinearity.

Review Manager (RevMan, version 8.1.1, The Cochrane Collaboration) software was used to conduct the meta-analysis. *P*≤0.05 was considered statistically significant. R version 4.3.1 (R Core Team, 2023) was used to model the dose-response relationship.

## Results

The PRISMA literature search flowchart is presented in Figure 1. Our electronic and manual search identified 1826 potential articles for screening. After excluding duplicate and irrelevant publications by screening titles and abstracts, 105 full-text articles were reviewed for eligibility. Sixty-seven articles were excluded at full-text review as they had different study designs, did not report BP outcome data, included other interventions, or included different routes of magnesium administration, had an intervention period <4 weeks, did not have a placebo or control group, were not provided in English language, included children, or full-text articles could not be retrieved. A total of 38 randomized controlled trials from published studies met our inclusion criteria.

### Characteristics of Studies

Table S2 presents the characteristics of the 38 eligible randomized controlled trials, including a total of 2709 participants, 1448 who received magnesium intervention and 1392 who received the placebo (131 participants were involved in crossover studies and received both magnesium intervention and placebo control). Participant ages ranged from 18 to 77 years. Most studies were conducted on both sexes, except for 2 that were restricted to men and 4 to women.

Five trials used a crossover design, whereas the remainder used a parallel design. Study intervention periods ranged from 4 to 24 weeks with a median duration of 12 weeks. All interventions used magnesium supplementation with elemental magnesium doses ranging from 82.3 to 637 mg, with a median dose of 365 mg. Twenty-seven studies included doses above the recommended dietary intake of magnesium for women (310–320 mg/d), and 9 exceeded the recommended dietary intake for men (400–420 mg/d).^22^ Magnesium supplementation included magnesium chloride (n=7), magnesium citrate (n=4), magnesium oxide (n=8), magnesium aspartate hydrochloride (n=8), magnesium pidolate (n=4), magnesium chelate (n=2), magnesium lactate (n=4), magnesium hydroxide (n=2), magnesium sulphate (n=2), and magnesium diclycine (n=1).

Seventeen trials included participants with hypertension, whereas 8 included participants with normotension. Of studies involving participants with hypertension, 9 included participants not taking BP-lowering medication within at least 1 month before the trial, whereas 6 included participants on BP-lowering medication. Eight trials included participants with hypomagnesemia (serum magnesium ≤0.74 mmol/L). Across these trials, BP measurements were conducted while participants were seated, supine, or standing, via 24-hour ambulatory BP or a combination of methods.

### Bias in Studies

Risk of bias assessments are presented in Table S3, including individual domain evaluation and overall risk of bias. Of all studies included in the meta-analysis, 9 were evaluated as low risk, 25 as some concern, and 4 as high risk. Most studies resulted in some concerns due to missing information about randomization and concealment methods.

Funnel plots for SBP and DBP are presented in Figures S1 and S2, respectively, and do not demonstrate asymmetry. The Egger test was also not statistically significant (SBP, *P*=0.52; DBP, *P*=0.97), indicating no evidence of asymmetry or publication bias.

### BP Outcomes

**Figure 2.**
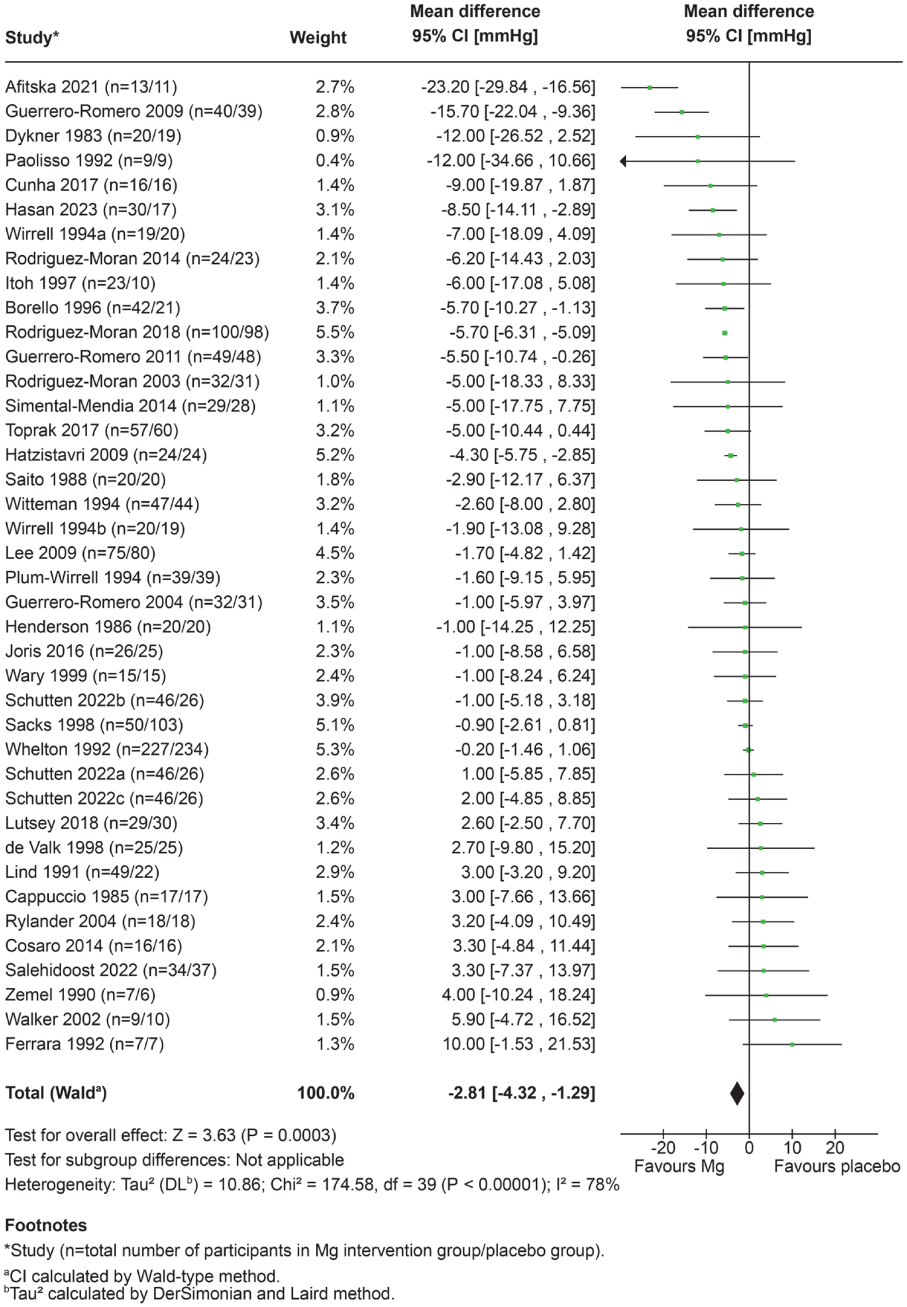
**Forest plot of change in systolic blood pressure (SBP; mm Hg) between magnesium intake and placebo groups, based on a random effects model.** All studies included (N=38).

**Figure 3.**
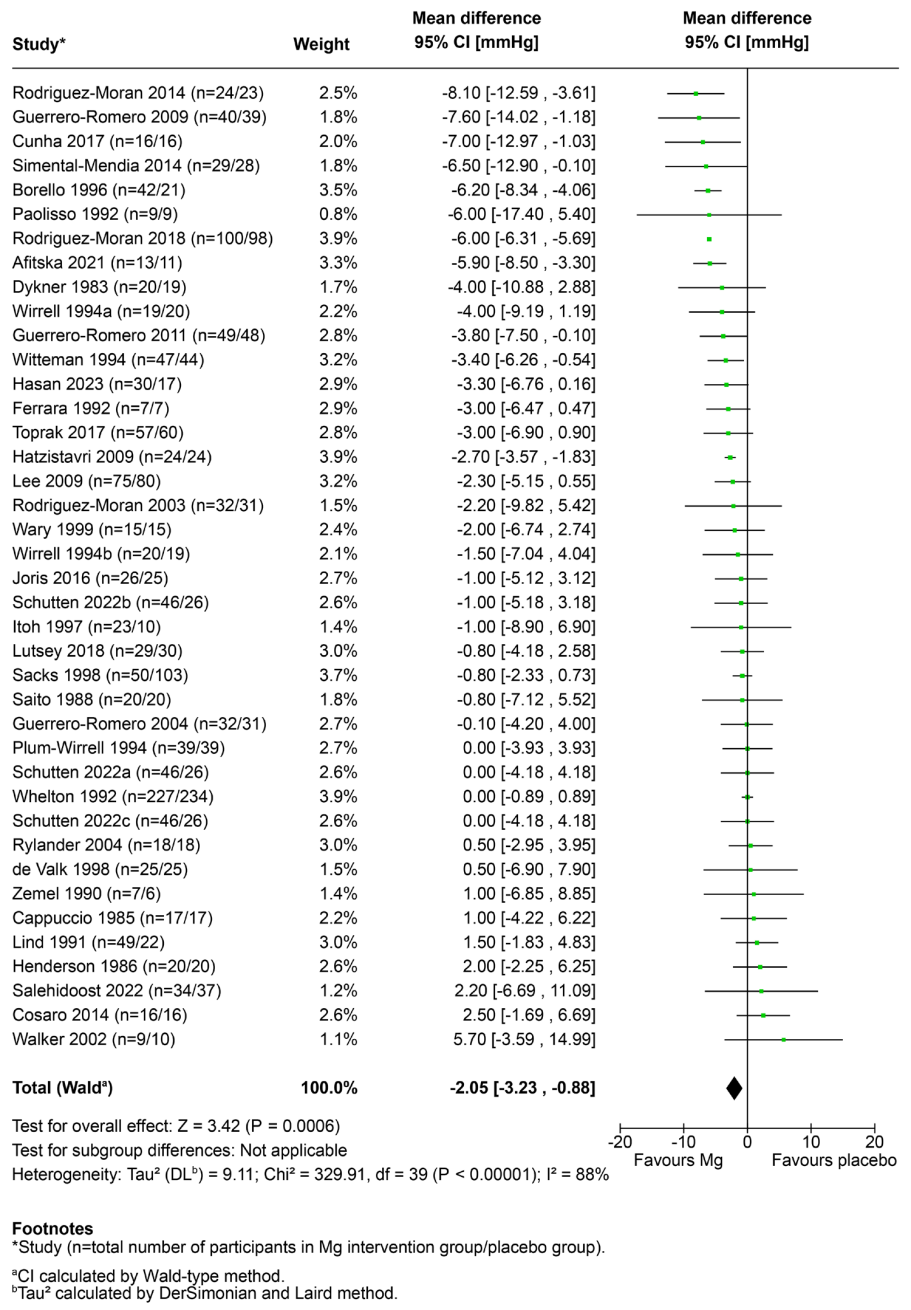
**Forest plot of change in diastolic blood pressure (DBP; mm Hg) between magnesium intake and placebo groups, based on a random effects model.** All studies included (N=38).

Forest plots comparing the mean changes in BP from baseline to the end of studies between magnesium intervention groups and control groups across all studies (N=38) are presented in Figure 2 for SBP and Figure 3 for DBP. The pooled results demonstrate that magnesium intake reduced SBP by −2.81 mm Hg (95% CI, −4.32 to −1.29; *P*<0.001) and DBP by −2.05 mm Hg (95% CI, −3.23 to −0.88; *P*<0.001). Heterogeneity for both cohorts was high (*I*^*2*^=78% for SBP and *I*^2^=88% for DBP).

### Analysis of Participants With Hypertension and Normotension

**Figure 4.**
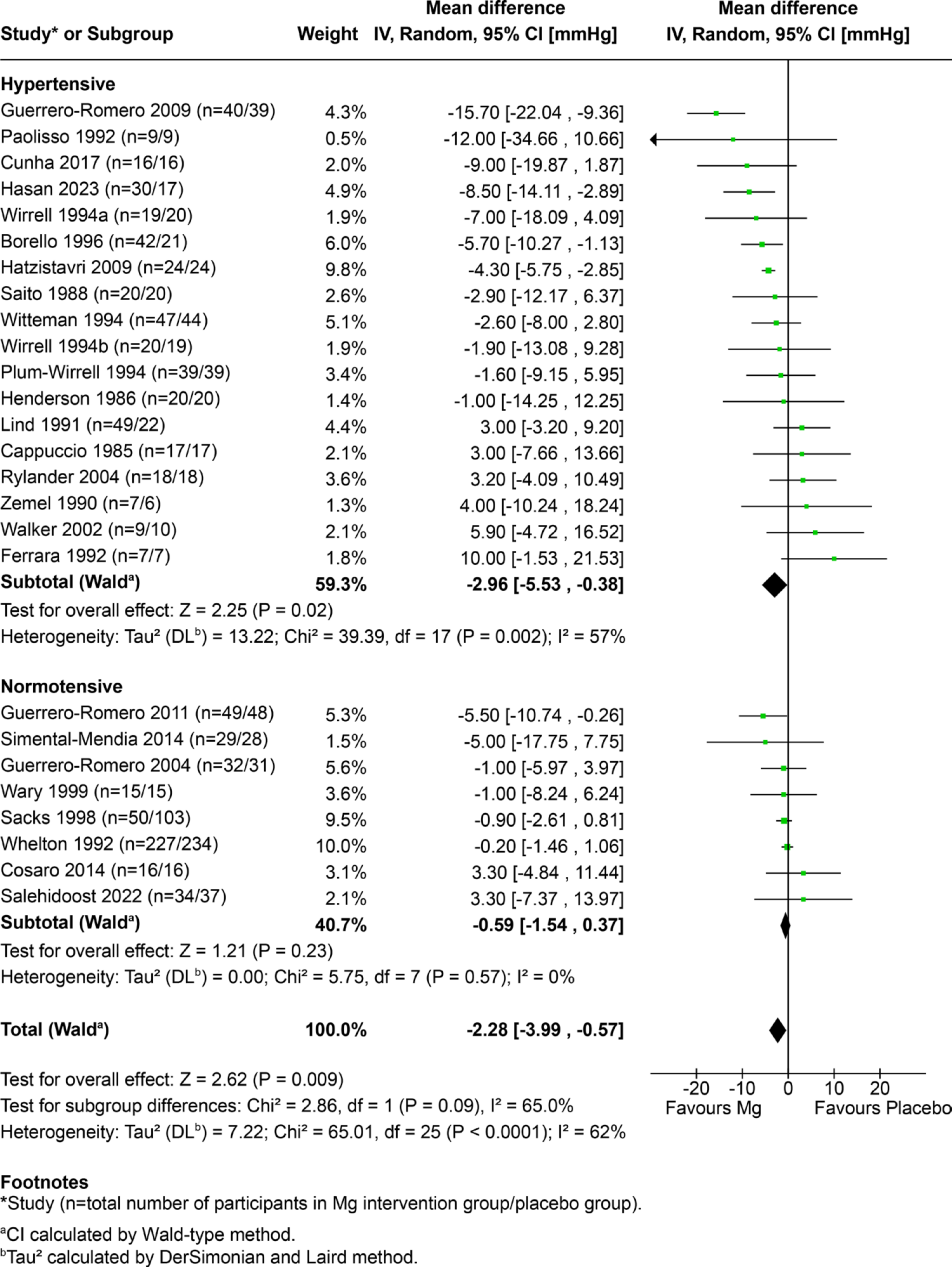
Forest plot of change in systolic blood pressure (SBP; mm Hg) among participants with hypertension (N=18) and normotension (N=8).

We undertook a subanalysis of studies reporting on participants with hypertension (N=17) compared with participants with normotension (N=8). These results are presented in Figure 4 for SBP and Figure S3 for DBP. In participants with hypertension, magnesium resulted in a SBP and DBP lowering of −2.96 mm Hg (95% CI, −5.53 to −0.38; *P*=0.02) and −2.10 mm Hg (95% CI, −3.44 to −0.77; *P*=0.002), respectively. Magnesium intervention did not produce significant reductions in SBP and DBP among participants with normotension.

The effect of magnesium intake on participants with hypertension was also assessed to compare those using BP-lowering medication (N=6) and untreated participants (N=9). Results of forest plots are reported in Figures S4 and S5. In participants with hypertension, magnesium intervention lowered SBP for those on BP-lowering medication by −7.68 mm Hg (95% CI, −12.67 to −2.69; *P*=0.003) but no changes were observed among those untreated, −0.90 mm Hg (95% CI, −3.94 to 2.14; *P*=0.56). Magnesium intake resulted in reductions in DBP among participants with hypertension on BP-lowering medication, −2.96 mm Hg (95% CI, −5.87 to −0.05; *P*=0.05) and without, −1.88 mm Hg (95% CI, −3.70 to −0.06;* P*=0.04) BP-lowering medication.

### Analysis According to Magnesium Status

Among participants with hypomagnesemia, magnesium intervention reduced SBP by −5.97 mm Hg (95% CI, −8.52 to −3.41;* P*<0.001). No significant changes in SBP were noted among participants with normomagnesemia (Figure S6). Results were also consistent with DBP in participants with hypomagnesemia (−4.75 mm Hg [95% CI, −6.59 to −2.92]; *P*<0.001) with no statistical significance seen for participants with normomagnesemia (Figure S7).

### Analysis According to the Body Position of BP Measurement

Most studies (N=22) measured BP of participants in a seated position, while 13 measured BP in supine and 2 via 24-hour ambulatory BP measurement. Of those who measured BP in supine, 7 also measured BP in standing. One study did not report a method for BP measurement.

In participants who had their BP measured in a seated position, magnesium intervention reduced SBP by −4.12 mm Hg (95% CI, −6.37 to −1.86; *P*<0.01) and DBP by −2.50 mm Hg (95% CI, −4.27 to −0.73; *P*=0.006). No significant changes in SBP and DBP were noted in participants who had their BP measured in supine or standing positions. Participants who had their BP measured by 24-hour ambulatory measurement demonstrated a reduction in DBP by −1.86 mm Hg (95% CI, −3.71 to −0.01; *P*=0.05) with no significant changes seen in SBP. Results are presented in Figure S8 for SBP and Figure S9 for DBP.

### Analysis According to the Study Design

Forest plots comparing the mean changes in SBP and DBP based on study design are presented in Figures S10 and S11. For studies that used a parallel study design, magnesium intervention reduced SBP by −3.03 mm Hg (95% CI, −4.64 to −1.41; *P*<0.001), although no significant changes in SBP were noted in crossover studies. Similar findings were seen for DBP, with parallel studies demonstrating a DBP reduction by −2.33 mm Hg (95% CI, −3.59 to −1.07; *P*<0.001) with no statistical significance seen for studies of crossover design.

### Analysis According to Magnesium Supplement Type

Forest plots comparing mean changes in SBP and DBP based on magnesium supplement type are presented in Figures S12 and S13, respectively. For studies that used magnesium chloride (N=7), magnesium intervention reduced SBP by –6.28 mm Hg (95% CI, –9.95 to –2.61;* P*<0.001) and reduced DBP by –5.01 mm Hg (95% CI, –7.00 to –3.03; *P*<0.001). Studies using magnesium aspartate hydrochloride (N=8) demonstrated a reduction in DBP with magnesium intervention by −1.82 mm Hg (95% CI, −3.51 to −0.13; *P*=0.03) but did not report a statistically significant reduction in SBP. No statistical significance was seen for studies using alternative magnesium supplementation types.

### Study Heterogeneity

We undertook a complete case multiple-adjusted mixed effects meta-regression to assess heterogeneity across studies by exploring how the proportion of male participants, mean age of participants, magnesium supplementation dose, intervention duration, and baseline SBP were associated with SBP and DBP effect sizes. In this meta-regression, SBP, sex, age, magnesium dose, intervention duration, and baseline SBP were not associated with effect size, and similarly for DBP. The meta-regression was conducted on 35 records, as 3 studies did not provide sex data, 2 did not provide age data, and 1 did not provide both sex and age data.

### Dose-Response Analysis

**Figure 5.**
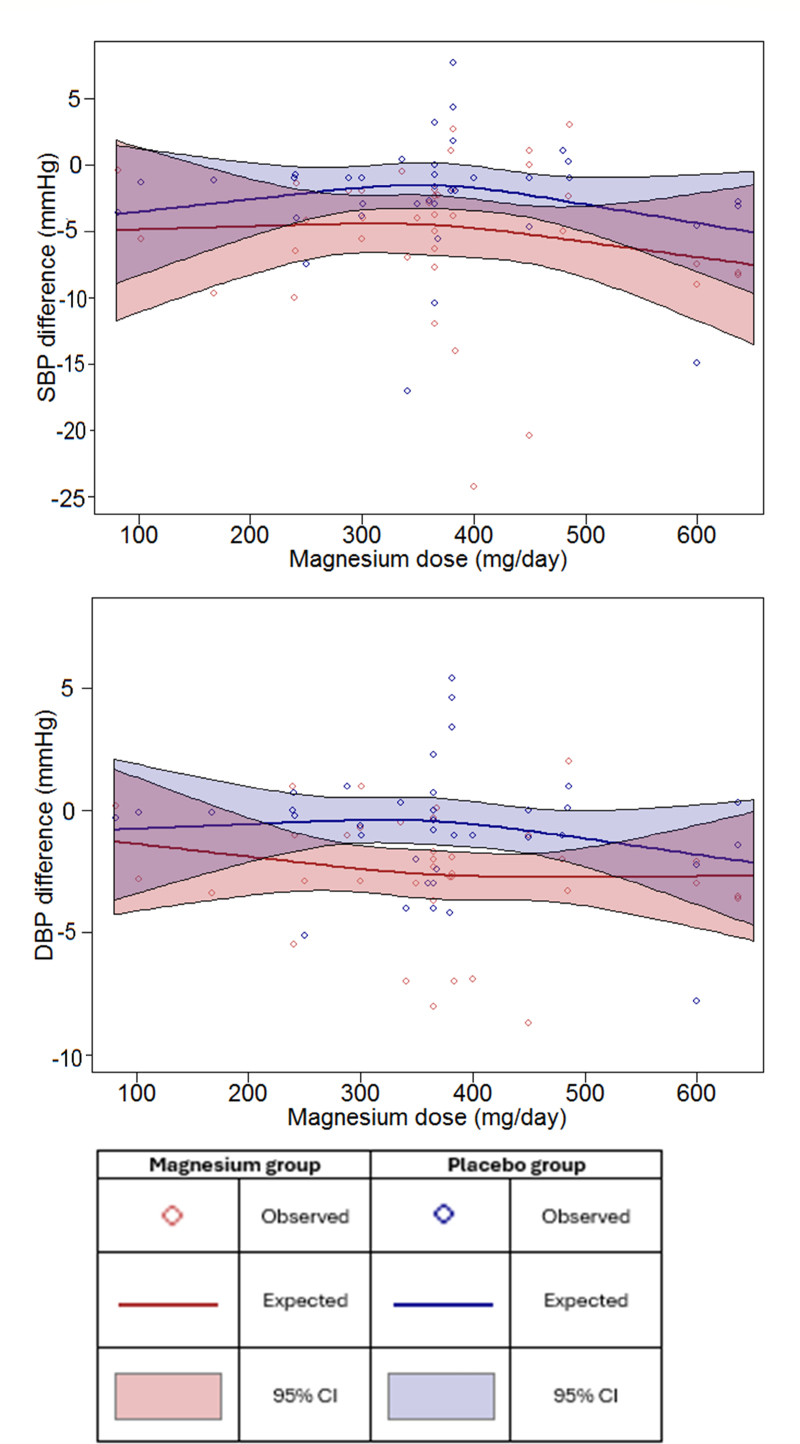
Dose-response relationship of magnesium and systolic blood pressure (SBP) and diastolic blood pressure (DBP) among magnesium intervention and placebo control groups (N=38).

Results for the dose-response meta-analysis demonstrating the relationship between magnesium intake and changes in SBP and DBP are presented in Figure 5. No statistically significant relationships were observed in either the linear or nonlinear models for both SBP (*P*=0.67 for linear and *P*=0.56 for nonlinear) and DBP (*P*=0.68 for linear and *P*=0.61 for nonlinear) in the magnesium intervention groups. Specifically, the change in SBP and DBP remained constant across increasing magnesium doses. Detailed results are reported in Table S4.

## Discussion

This review of 38 randomized controlled trials involving 2709 participants found that magnesium supplementation with a median intake of 365 mg/d for a median duration of 12 weeks led to a significant reduction in both SBP and DBP (−2.84 and −2.10 mm Hg, respectively). Although these reductions in BP were consistent, on a clinical level, the BP-lowering effect is modest. Despite evidence that magnesium intake does decrease SBP and DBP, we did not find a dose-response relationship between magnesium intake and BP, as others have reported.^23^

Past analyses have attempted to determine dose-response relationships through a subanalysis of doses. Kass et al^24^ compared magnesium doses of <370 mg and ≥370 mg, and found that magnesium supplementation if ≥370 mg was associated with greater efficacy of magnesium supplementation, while a smaller effect estimates and larger variations in BP were noted with doses <370 mg. Rosanoff et al^25^ found that while ≥240 mg/d of magnesium was effective at reducing BP in treated but uncontrolled hypertensive individuals, a dose of ≥600 mg/d was required to lower BP among untreated hypertensive participants. However, due to the heterogeneity between studies and the narrow magnesium doses included in studies, our analysis suggests that further exploration of a dose-response relationship in subpopulations, along with the assessment of studies trialing a higher magnesium dose, may be required to better inform and guide the therapeutic benefits of magnesium.

Pooled results from 17 studies in participants with hypertension showed a −2.96/−2.10 mm Hg reduction in SBP and DBP. The results demonstrated that while untreated hypertensive participants achieved significant reductions in DBP (1.88 mm Hg), only those on BP-lowering medication achieved significant reductions in both SBP and DBP, with reductions of −7.68 and −2.96 mm Hg, respectively, at the magnesium dose provided. The reduction, particularly in SBP among this population, is clinically important, with a recent meta-analysis finding that a 5 mm Hg reduction in SBP reduced the risk of major cardiovascular events by ≈10%.^26^

Although the benefits of magnesium were demonstrated in participants with hypertension on BP-lowering medication, the meta-analysis found that magnesium intake was not effective at reducing SBP and DBP among participants with normotension. This builds on the categorization systematic review of 49 studies by Rosanoff et al^25^ that found similar results, including that magnesium therapy decreased BP in treated hypertensive participants, although no changes were observed in normotensive individuals. Unlike the current meta-analysis, the previous review did not quantifiably determine BP changes.

It is suggested that systemic magnesium depletion promotes increased BP in patients with hypertension through increasing vascular and sympathetic tone, altering sodium and potassium handling, enhancing the inflammatory process, and impairing vascular cell metabolism and dysfunction.^11^ Diuretics, prescribed as a first-line pharmacological therapy for hypertension, have been shown to exacerbate magnesium depletion among patients with hypertension through increased renal magnesium loss. Of the 6 studies included in this review that assessed patients with hypertension on BP-lowering medication, 4 studies (Paolisso et al, Saito et al, Guerrero-Romero et al, and Henderson et al) included participants on diuretics (n=66). This may explain why participants with hypertension taking BP-lowering medication demonstrated greater BP benefits of −7.68/−2.96 mm Hg when receiving magnesium treatment.^2,11,15^

The impact of magnesium depletion on BP may also demonstrate why BP reductions were identified among participants with hypomagnesemia and not those with normomagnesemia.^25^ This is supported by Zhang et al,^16^ who conducted a dose-response analysis of 34 studies and found that a 0.1 mmol/L increase in serum magnesium was associated with a 2.26 mm Hg reduction in DBP. This association was not significant with SBP.^16^

This review demonstrated that magnesium chloride and magnesium aspartate hydrochloride have the potential to yield greater BP reductions compared with alternative magnesium supplement types. However, these results must be interpreted with caution due to the small number of studies for some magnesium supplement types. Of the studies using magnesium chloride as their supplement intervention, all were conducted on participants with hypomagnesemia, and thus, the results may be confounded by the effects of magnesium depletion. These results also conflict with previous studies that have demonstrated organic magnesium compounds are better absorbed than inorganic compounds ^15^

Interestingly, we only found significant changes in SBP and DBP when BP was taken in a seated body position (−4.12/−2.50 mm Hg) and not in standing (0.02/0.60 mm Hg) or supine positions (−0.73/−1.47 mm Hg). This may be important when considering the impact of magnesium on orthostatic hypotension, which is common among those with uncontrolled hypertension and is an independent risk factor for cardiovascular comorbidities and mortality.^27^ Standing BP may, therefore, be an important measure to consider when developing therapeutic treatments, and further studies assessing BP in different body positions may be needed to determine whether these changes are important.

### Limitations

Several limitations should be considered when interpreting our findings. A major limitation in most studies was the lack of data on dietary intake and, therefore, the overall magnesium intake of participants. Moreover, magnesium preparations have different pharmacokinetic and pharmacodynamic features, which influence bioavailability.^28^ Absorption of organic magnesium compounds is better than absorption of inorganic compounds. Significant heterogeneity was also present across studies, and although this was reduced when we undertook subgroup analyses, sources of heterogeneity were not identified through additional meta-regression of participant and study characteristics. Differences in study designs, BP measurement techniques, magnesium supplement composition, baseline clinical status of participants, along with small sample sizes, may have contributed, making it difficult to draw conclusions.

Most studies used a magnesium dose between 300 and 500 mg and yielded a wide range of BP responses. This limited the ability to perform a dose-response analysis across a wide range of doses to undertake a more comprehensive dose-response analysis. Dose-response analysis was also undertaken for magnesium dose, but was not completed for the dose of intervention duration, which may have increased the robustness of the analysis.

Hypomagnesemia and normomagnesemia categories were determined by studies based on reported categories of serum magnesium. However, <1% of magnesium in the body is found in the blood, and therefore, serum magnesium concentrations may underestimate depleted intracellular stores and magnesium deficiency.^15^ For a more accurate evaluation of magnesium status, total dietary magnesium and urinary losses should be considered.^15^

### Perspectives

This meta-analysis strengthens the evidence to support the benefits of oral magnesium intake for the reduction in SBP and DBP, with potentially greater benefit for patients with hypertension using BP-lowering medication, and for patients with hypomagnesemia. We found no dose-response relationship. Importantly, the absence of a significant effect in normotensive populations and the lack of a clear dose-response relationship underscore the complexity of magnesium’s physiological influence and the need for further research. The high heterogeneity across studies emphasizes the necessity for future large-scale, rigorously designed randomized controlled trials to elucidate optimal dosing thresholds and identify responsive subpopulations. These findings contribute to the growing body of evidence supporting personalized nutritional approaches in hypertension management and call for integration of micronutrient assessment in clinical practice, particularly where the hypertension burden is high.

## ARTICLE INFORMATION

### Sources of Funding

A.E. Schutte is funded by an Investigator Grant from the National Health and Medical Research Council of Australia (APP2017504).

### Disclosures

A.E. Schutte has received speaker honoraria from Servier, Abbott, Sanofi, AstraZeneca, Medtronic, Omron, and Aktiia and serves on scientific advisory boards for Medtronic, Roche/Alnylam, AstraZeneca, Servier, SiSU Health, and Sky Labs. The other authors report no conflicts.

### Supplemental Material

Tables S1–S4

Figures S1–S13

References 29–66

## Supplementary Material



## References

[1] GBD 2019 Risk Factors Collaborators. Global burden of 87 risk factors in 204 countries and territories, 1990-2019: a systematic analysis for the Global Burden of Disease Study 2019. Lancet. 2020;396:1223–1249. doi: 10.1016/S0140-6736(20)30752-2 33069327 10.1016/S0140-6736(20)30752-2 PMC7566194

[2] Unger T Borghi C Charchar F Khan NA Poulter NR Prabhakaran D Ramirez A Schlaich M Stergiou GS Tomaszewski M. 2020 International Society of Hypertension Global Hypertension Practice Guidelines. Hypertension. 2020;75:1334–1357. doi: 10.1161/HYPERTENSIONAHA.120.15026 32370572 10.1161/HYPERTENSIONAHA.120.15026

[3] Green RC Marklund M Anderson CA Covv KL Dalcin AT Henry M Appel LJ. Potassium-enriched salt substitutes as a means to lower blood pressure: benefits and risks. Hypertension. 2020;75:266–274. doi: 10.1161/hypertensionaha.119.13241 31838902 10.1161/HYPERTENSIONAHA.119.13241

[4] Filippou CD Tsioufis CP Thomopoulos CG Mihas CC Dimitriadis KS Sotiropoulou LI Chrysochoou C Nihoyannapoulos PI Tousoulis DM. Dietary Approaches to Stop Hypertension (DASH) diet and blood pressure reduction in adults with and without hypertension: a systematic review and meta-analysis of randomized controlled trials. Adv Nutr. 2020;11:1150–1160. doi: 10.1093/advances/nmaa041 32330233 10.1093/advances/nmaa041 PMC7490167

[5] Sacks FM Svetkey LP Vollmer WM Appel LJ Bray GA Harsha D Obarzanek E Conlin PR Miller ER Simons-Morton DG. Effects on blood pressure of reduced dietary sodium and Dietary Approaches to Stop Hypertension (DASH) diet. DASH-Sodium Collaborative Research Group. N Engl J Med. 2001;344:3–10. doi: 10.1056/nejm200101043440101 11136953 10.1056/NEJM200101043440101

[6] He FJ Macgregor GA. Effect of longer term modest salt reduction on blood pressure: cochrane systematic review and meta-analysis or randomised trials. BMJ. 2013;346:f1325. doi: 10.1136/bmj.f1325 23558162 10.1136/bmj.f1325

[7] He FJ Tan M Ma Y MacGregor GA. Salt reduction to prevent hypertension and cardiovascular disease: JACC state-of-the-art review. J Am Coll Cardiol. 2020;75:632–647. doi: 10.1016/j.jacc.2019.11.055 32057379 10.1016/j.jacc.2019.11.055

[8] Filippini T Naska A Kasdagli M-I Torres D Lopes C Carvalho C Moreira P Malavolti M Orsini N Whelton PK. Potassium intake and blood pressure: a dose-response meta-analysis of randomized controlled trial. J Am Heart Assoc. 2020;9:e015719. doi: 10.1161/JAHA.119.015719 32500831 10.1161/JAHA.119.015719 PMC7429027

[9] Kavanaugh C. RE: petition for a qualified health claim for magnesium and reduced risk of high blood pressure (hypertension) (Docket No. FDA-2016-Q-3770). Centre for Food Safety and Applied Nutrition: US. 2022

[10] de Baaij JHF Hoenderop JGJ Bindels RJM. Magnesium in man: implications for health and disease. Physiol Rev. 2015;95:1–46. doi: 10.1152/physrev.00012.2014 25540137 10.1152/physrev.00012.2014

[11] Alshanableh Z Ray EC. Magnesium in hypertension: mechanisms and clinical implications. Front Physiol. 2024;15:1363975. doi: 10.3389/fphys.2024.1363975 38665599 10.3389/fphys.2024.1363975 PMC11044701

[12] Houston M. The role of magnesium in hypertension and cardiovascular disease. J Clin Hypertens (Greenwich). 2011;13:843–847. doi: 10.1111/j.1751-7176.2011.00538.x 22051430 10.1111/j.1751-7176.2011.00538.x PMC8108907

[13] Dibaba DT Xun P Song Y Rosanoff A Shechter M He K. The effect of magnesium supplementation on blood pressure in individuals with insual resistance, prediabetes, or noncommunicable chronic diseases: a meta-analysis of randomized controlled trials. Am J Clin Nutr. 2017;106:921–929. doi: 10.3945/ajcn.117.155291 28724644 10.3945/ajcn.117.155291 PMC5573024

[14] Schutten JC Joosten MM Borst MH Bakker SJL. Magnesium and blood pressure: a physiology-based approach. Adv Chronic Kidney Dis. 2018;25:144–250. doi: 10.1053/j.ackd.2017.12.003 10.1053/j.ackd.2017.12.003 29793663

[15] Touyz RM Baaiji JHF Joenderop JGJ. Magnesium disorders. N Engl J Med. 2024;390:1998–2009. doi: 10.1056/NEJMra1510603 38838313 10.1056/NEJMra1510603

[16] Zhang X Li Y Gobbo LCD Rosanoff A Wang J Zhang W Song Y. Effects of magnesium supplementation on blood pressure: a meta-analysis of randomized double-blind placebo-controlled trials. Hypertension. 2016;68:324. doi: 10.1161/HYPERTENSIONAHA.116.07664 27402922 10.1161/HYPERTENSIONAHA.116.07664

[17] Jee SH Miller ER Guallar E Singh VK Appel LJ Klag MJ. The effect of magnesium supplementation on blood pressure: a meta-analysis of randomized clinical trials. Am J Hypertens. 2002;15:691–696. doi: 10.1016/s0895-7061(02)02964-3 12160191 10.1016/s0895-7061(02)02964-3

[18] Dickinson HO Nicolson D Campbell F Cook JV Beyer FR Ford GA Mason J. Magensium supplementation for the management of primary hypertension in adults. Cochrane Database Syst Rev. 2006;(3):CD004640. doi: 10.1002/14651858.CD004640.pub2 16625609 10.1002/14651858.CD004639.pub2 PMC12410052

[19] Higgins J Thomas J Chandler J Cumpston M Li T Page M Welch V. Cochrane handbook for systematic reviews of interventions. Version 6.5 ed. John Wiley & Sons; 2024

[20] Fu R Vandemeer BW Shamliyan TA O’Neil ME Jazdi F Fox SH Morton SC. Handling continuous outcomes in quantitative synthesis. In: Methods guide for effectiveness and comparative effectiveness reviews. Rockville: Agency for Healthare Research and Quality; 2008 24006546

[21] Sterne JAC Savovic J Page MJ Elbers RG Blencowe NS Boutron I Cates CJ Cheng HY Corbett MS Eldrige SM. RoB 2: a revised tool for assessing risk of bias in randomised trials. BMJ. 2019;366:l4898. doi: 10.1136/bmj.l4898 31462531 10.1136/bmj.l4898

[22] National Health and Medical Research Council. Nutrient reference values for australia and New Zealand: magnesium. Commonwealth of Australia:Canberra; 2006:193–200

[23] Han H Fang X Wei X Liu Y Jin Z Chen Q Fan Z Aaseth J Hiyoshi A He J. Dose-response relationship between dietary magnesium intake, serum magnesium concentration and risk of hypertension: a systematic review and meta-analysis of prospective cohort studies. Nutr J. 2017;16:26. doi: 10.1186/s12937-017-0247-4 28476161 10.1186/s12937-017-0247-4 PMC5420140

[24] Kass L Weekes J Carpenter L. Effect of magnesium supplementation on blood pressure: a meta-analysis. Eur J Clin Nutr. 2012;66:411–418. doi: 10.1038/ejcn.2012.4 22318649 10.1038/ejcn.2012.4

[25] Rosanoff A Costello RB Johnson GH. Effectively prescribing oral magnesium therapy for hypertension: a categorized systematic review of 49 clinical trials. Nutrients. 2021;13:195. doi: 10.3390/nu13010195 33435187 10.3390/nu13010195 PMC7827637

[26] The Blood Pressure Lowering Treatment Trialists’ Collaboration. Pharmacological blood pressure lowering for primary and secondary prevention of cardiovascular disease across different levels of blood pressure: an individual participant level data meta-analysis. Lancet. 2021;397:1625–1636. doi: 10.1016/S0140-6736(21)00590-0 33933205 10.1016/S0140-6736(21)00590-0 PMC8102467

[27] Juraschek SP Cortez MM Flack JM Ghazi L Kenny RA Rahman M Spikes T Shibao CA Biaggioni I; American Heart Association Council on Hypertension. Orthostatic hypotension in adults with hypertension: a scientific statement from the American Heart Association. Hypertension. 2024;81:e16–e30. doi: 10.1161/HYP.0000000000000236 38205630 10.1161/HYP.0000000000000236 PMC11067441

[28] Uysal N Kizildag S Yuce Z Guvendi G Kandis S Koc B Karakilic A Camsari UM Ates M. Timeline (bioavailability) of magnesium compounds in hours: which magnesium compound works best? Biol Trace Elem Res. 2019;187:128–136. doi: 10.1007/s12011-018-1351-9 29679349 10.1007/s12011-018-1351-9

[29] Afitska K Clavel J Kisters K Vormann J Werner T. Magnesium citrate supplementation decreased blood pressure and HbA1c in normomagnesemic subjects with metabolic syndrome: a 12-week, placebo-controlled, double-blinded pilot trial. Magnes Res. 2021;34:130–139. doi: 10.1684/mrh.2021.0489 34859788 10.1684/mrh.2021.0489

[30] Borello G Mastroroberto P Curcio F Chello M Zofrea S Mazza ML. The effects of magnesium oxide on mild essential hypertension and quality of life. Curr Ther Res. 1996;57:767–774. doi: 10.1016/S0011-393X(96)80082-8

[31] Cappuccio FP Markandu ND Beynon GW Shore AC Sampson B MacGregor GA. Lack of effect of oral magnesium on high blood pressure: a double blind study. Br Med J (Clin Res Ed). 1985;291:235–238. doi: 10.1136/bmj.291.6490.235 10.1136/bmj.291.6490.235 PMC1416881 3926135

[32] Cosaro E Bonafini S Montagnana M Danese E Trettene MS Minuz P Delva P Fava C. Effects of magnesium supplements on blood pressure, endothelial function and metabolic parameters in healthy young men with a family history of metabolic syndrome. Nutr Metab Cardiovasc Dis. 2014;24:1213–1220. doi: 10.1016/j.numecd.2014.05.010 24984823 10.1016/j.numecd.2014.05.010

[33] Cunha AR D’El-Rei J Medeiros F Umbelino B Oigman W Touyz RM Neves MF. Oral magnesium supplementation improves endothelial function and attenuates subclinical atherosclerosis in thiazide-treated hypertensive women. J Hypertens. 2017;35:89–97. doi: 10.1097/HJH.0000000000001129 27759579 10.1097/HJH.0000000000001129

[34] de Valk HW Verkaaik R van Rijn HJ Geerdink RA Struyvenberg A. Oral magnesium supplementation in insulin-requiring type 2 diabetic patients. Diabet Med. 1998;15:503–507. doi: 10.1002/(SICI)1096-9136(199806)15:6<503::AID-DIA596>3.0.CO;2-M 9632126 10.1002/(SICI)1096-9136(199806)15:6<503::AID-DIA596>3.0.CO;2-M

[35] Dyckner T Wester PO. Effect of magnesium on blood pressure. Br Med J (Clin Res Ed). 1983;286:1847–1849. doi: 10.1136/bmj.286.6381.1847 10.1136/bmj.286.6381.1847 PMC1547774 6407598

[36] Ferrara LA Iannuzzi R Castaldo A Iannuzzi A Dellorusso A Mancini M. Long-term magnesium supplementation in essential-hypertension. Cardiology. 1992;81:25–33. doi: 10.1159/000175772 1477853 10.1159/000175772

[37] Guerrero-Romero F Tamez-Perez H Gonzalez-Gonzalez G Salinas-Martinez A Montes-Villarreal J Trevino-Ortiz J Rodriguez-Moran M. Oral Magnesium supplementation improves insulin sensitivity in non-diabetic subjects with insulin resistance. A double-blind placebo-controlled randomized trial. Diabetes Metab. 2004;30:253–258. doi: 10.1016/s1262-3636(07)70116-7 15223977 10.1016/s1262-3636(07)70116-7

[38] Guerrero-Romero F Rodríguez-Morán M. The effect of lowering blood pressure by magnesium supplementation in diabetic hypertensive adults with low serum magnesium levels: a randomized, double-blind, placebo-controlled clinical trial. J Hum Hypertens. 2009;23:245–251. doi: 10.1038/jhh.2008.129 19020533 10.1038/jhh.2008.129

[39] Guerrero-Romero F Rodriguez-Moran M. Magnesium improves the beta-cell function to compensate variation of insulin sensitivity: double-blind, randomized clinical trial. Eur J Clin Invest. 2011;41:405–410. doi: 10.1111/j.1365-2362.2010.02422.x 21241290 10.1111/j.1365-2362.2010.02422.x

[40] Hasan EK Mshimesh BAR Rzoqi SS Aziz LSA Khazaal FAK Marzooq AA Abudlqader EH. Effect of magensium l-lactate supplement on blood pressure and corrected QT interval in a sample of Iraqui women with metabolic syndrome. Wiad Lek. 2023;76:360–369. doi: 10.36740/WLek202302117 37010174 10.36740/WLek202302117

[41] Hatzistavri LS Sarafidis PA Georgianos PI Tziolas IM Aroditis CP Zebekakis PE Pikilidou MI Lasaridis AN. Oral magnesium supplementation reduces ambulatory blood pressure in patients with mild hypertension. Am J Hypertens. 2009;22:1070–1075. doi: 10.1038/ajh.2009.126 19617879 10.1038/ajh.2009.126

[42] Henderson DG Schierup J Schødt T. Effect of magnesium supplementation on blood pressure and electrolyte concentrations in hypertensive patients receiving long term diuretic treatment. Br Med J (Clin Res Ed). 1986;293:664–665. doi: 10.1136/bmj.293.6548.664 10.1136/bmj.293.6548.664 PMC1341513 3092972

[43] Itoh K Kawasaka T Nakamura M. The effects of high oral magnesium supplementation on blood pressure, serum lipids and related variables in apparently healthy Japanese subjects. Br J Nutr. 1997;78:737–750. doi: 10.1079/bjn19970191 9389897 10.1079/bjn19970191

[44] Joris PJ Plat J Bakker SJ Mensink RP. Long-term magnesium supplementation improves arterial stiffness in overweight and obese adults: results of a randomized, double-blind, placebo-controlled intervention trial. Am J Clin Nutr. 2016;103:1260–1266. doi: 10.3945/ajcn.116.131466 27053384 10.3945/ajcn.116.131466

[45] Lee S Park HK Son SP Lee CW Kim IJ Kim HJ. Effects of oral magnesium supplementation on insulin sensitivity and blood pressure in normo-magnesemic nondiabetic overweight Korean adults. Nutr Metab Cardiovasc Dis. 2009;19:781–788. doi: 10.1016/j.numecd.2009.01.002 19359148 10.1016/j.numecd.2009.01.002

[46] Lind L Lithell H Pollare T Ljunghall S. Blood pressure response during long-term treatment with magnesium is dependent on magnesium status. A double-blind, placebo-controlled study in essential hypertension and in subjects with high-normal blood pressure. Am J Hypertens. 1991;4:674–679. doi: 10.1093/ajh/4.8.674 1930849 10.1093/ajh/4.8.674

[47] Lutsey PL Chen LY Eaton A Jaeb M rudser KD Neaton JD Alonso A. A pilot randomized trial of oral magnesium supplementation on supraventricular arrhythmias. Nutrients. 2018;10:884. doi: 10.3390/nu10070884 29996476 10.3390/nu10070884 PMC6073799

[48] Mooren FC Krüger K Völker K Golf SW Wadepuhl M Kraus A. Oral magnesium supplementation reduces insulin resistance in non-diabetic subjects - a double-blind, placebo-controlled, randomized trial. Diabetes Obes Metab. 2011;13:281–284. doi: 10.1111/j.1463-1326.2010.01332.x 21205110 10.1111/j.1463-1326.2010.01332.x

[49] Paolisso G De Maro G Cozzolino D Salvatore T D’Amore A Lama D Varricchio M D’Onofrio F. Chronic magnesium administration enhances oxidative glucose metabolism in thiazide treated hypertensive patients. Am J Hypertens. 1992;5:681–686. doi: 10.1093/ajh/5.10.681 1418829 10.1093/ajh/5.10.681

[50] Plum-Wirell M Stegmayr BG Wester PO. Nutritional magnesium supplementation does not change blood pressure nor serum or muscle potassium and magnesium in untreated hypertension. A double-blind crossover study. Magnes Res. 1994;7:277–283. 7786691

[51] Rodríguez-Moran M Guerrero-Romero F. Oral magnesium supplementation improves the metabolic profile of metabolically obese, normal-weight individuals: a randomized double-blind placebo-controlled trial. Arch Med Res. 2014;45:388–393. doi: 10.1016/j.arcmed.2014.05.003 24830937 10.1016/j.arcmed.2014.05.003

[52] Rodríguez-Morán M Simental-Mendía LE Gamboa-Gómez CI Guerrero-Romero F. Oral magnesium supplementation and metabolic syndrome: a randomized double-blind placebo-controlled clinical trial. Adv Chronic Kidney Dis. 2018;25:261–266. doi: 10.1053/j.ackd.2018.02.011 29793665 10.1053/j.ackd.2018.02.011

[53] Rodríguez-Morán M Guerrero-Romero F. Oral magnesium supplementation improves insulin sensitivity and metabolic control in type 2 diabetic subjects: a randomized double-blind controlled trial. Diabetes Care. 2003;26:1147–1152. doi: 10.2337/diacare.26.4.1147 12663588 10.2337/diacare.26.4.1147

[54] Rylander R Arnaud MJ. Mineral water intake reduces blood pressure among subjects with low urinary magnesium and calcium levels. BMC Public Health. 2004;4:56. doi: 10.1186/1471-2458-4-56 15571635 10.1186/1471-2458-4-56 PMC535900

[55] Sacks FM Willett WC Smith A Brown LE Rosner B Moore TJ. Effect on blood pressure of potassium, calcium, and magnesium in women with low habitual intake. Hypertension. 1998;31:131–138. doi: 10.1161/01.hyp.31.1.131 9449404 10.1161/01.hyp.31.1.131

[56] Saito K Hattori K Omatsu T Hirouchi H Sano H Fukuzaki H. Effects of oral magnesium on blood pressure and red cell sodium transport in patients receiving long-term thiazide diuretics for hypertension. Am J Hypertens. 1988;1:71S–74S. doi: 10.1093/ajh/1.3.71s 3415812 10.1093/ajh/1.3.71s

[57] Salehidoost R Boroujani GT Feizi A. Effect of oral magnesium supplement on cardiometabolic markers in people with prediabetes: a double blind randomized controlled clinical trial. Sci Rep. 2022;12:18209. doi: 10.1038/s41598-022-20277-6 36307427 10.1038/s41598-022-20277-6 PMC9616938

[58] Schutten JC Joris PJ Groendijk I Eelderink C Groothof D van der Veen Y Westerhuis R Goorman F Danel RM de Borst MH. Effects of magnesium citrate, magnesium oxide, and magnesium sulfate supplementation on arterial stiffness: a randomized, double-blind, placebo-controlled intervention trial. J Am Heart Assoc. 2022;11:e021783. doi: 10.1161/JAHA.121.021783 35253448 10.1161/JAHA.121.021783 PMC9075273

[59] Simental-Mendia LE Rodriguez-Moran M Guerrero-Romero F. Oral magnesium supplementation decreases c-reactive protein levels in subjects with prediabetes and hypomagnesemia: a clinical randomized double-blind placebo-controlled trial. Arch Med Res. 2014;45:325–330. doi: 10.1016/j.arcmed.2014.04.006 24814039 10.1016/j.arcmed.2014.04.006

[60] Toprak O Kurt H Sari Y Sarkis C Us H Kirik A. Magnesium replacement improves the metabolic profile in obese and pre-diabetic patients with mild-to-moderate chronic kidney disease: a 3-month, randomised, double-blind, placebo-controlled study. Kidney Blood Press Res. 2017;42:33–42. doi: 10.1159/000468530 28297698 10.1159/000468530

[61] Walker AF Marakis G Morris AP Robinson PA. Promising hypotensive effect of hawthorn extract: a randomized double-blind pilot study of mild, essential hypertension. Phytother Res. 2002;16:48–54. doi: 10.1002/ptr.947 11807965 10.1002/ptr.947

[62] Wary C Brillault-Salvat C Bloch G Leroy-Willig A Roumenov D Grognet J-M Leclerc JH Carlier PG. Effect of chronic magnesium supplementation on magnesium distribution in healthy volunteers evaluated by P-NMRS and ion selective electrodes. Br J Clin Pharmacol. 1999;48:655–662. doi: 10.1046/j.1365-2125.1999.00063.x 10594466 10.1046/j.1365-2125.1999.00063.x PMC2014351

[63] The Trials of Hypertension Prevention (TOHP) Collaborative Research Group. The effects of nonpharmacologic interventions on blood pressure of persons with high normal levels. JAMA. 1992;267:1213–1220. doi: 10.1001/jama.1992.03480090061028 1586398 10.1001/jama.1992.03480090061028

[64] Wirell MP Wester PO Stegmayr BG. Nutritional dose of magnesium in hypertensive patients on beta blockers lowers systolic blood pressure: a double-blind, cross-over study. J Intern Med. 1994;236:189–195. doi: 10.1111/j.1365-2796.1994.tb01282.x 7913949 10.1111/j.1365-2796.1994.tb01282.x

[65] Witteman JC Grobbee DE Derkx FH Bouillon R de Bruijn AM Hofman A. Reduction of blood pressure with oral magnesium supplementation in women with mild to moderate hypertension. Am J Clin Nutr. 1994;60:129–135. doi: 10.1093/ajcn/60.1.129 8017327 10.1093/ajcn/60.1.129

[66] Zemel PC Zemel MB Urberg M Douglas FL Geiser R Sowers JR. Metabolic and hemodynamic effects of magnesium supplementation in patients with essential hypertension. Am J Clin Nutr. 1990;51:665–669. doi: 10.1093/ajcn/51.4.665 2181860 10.1093/ajcn/51.4.665

