# Supplementary material for: Magnesium Supplementation and Blood Pressure: A Systematic Review and Meta-Analysis of Randomized Controlled Trials
Source: Hypertension. 2025 Sep 26;82(11):1844–56. doi: 10.1161/HYPERTENSIONAHA.125.25129 (PMC12529988; doi:10.1161/HYPERTENSIONAHA.125.25129)
Supplement: Supplementary file 1 [file hyp-82-1844-s001.docx]

**SUPPLEMENTARY MATERIAL**

**Magnesium supplementation and blood pressure:
A systematic review and meta-analysis of randomized controlled trials**

Zoe Argeros^1^, Xiaoye Xu^1,2^, Buna Bhandari^3,4^, Katie Harris^2^, Rhian M Touyz^5^, Aletta E Schutte^1,2^

**Affiliations:**

^1^School of Population Health, University of New South Wales, Sydney, NSW, Australia

^2^ The George Institute for Global Health, Sydney, NSW, Australia

^3^ Department of Global Health and Population, Harvard T H Chan School of Public Health, Boston, MA, USA

^4^Florida State University College of Nursing, Tallahassee, Florida, USA

^5^Research Institute of McGill University Health Centre, McGill University, Department of medicine and Department of family medicine, Montreal, Quebec, Canada.

**Short Title:** Magnesium intake and blood pressure

**Correspondence:** Professor AE Schutte, School of Population Health, University of New South Wales Sydney, High St, Kensington, NSW 2052, Australia.

Email: a.schutte@unsw.edu.au

**Supplementary** **Table S1.** Search strategies for online databases

| **Database** | **Search Strategy** |
| --- | --- |
| PubMed/MEDLINE | #1"Magnesium"[Mesh]  #2 magnesium[Title/Abstract] OR mg[Title/Abstract]  #3 #1 OR #2  #4 "Blood Pressure"[Mesh] OR "Hypertension"[Mesh]  #5 "blood pressure"[Title/Abstract] OR hyperten*[Title/Abstract] OR systolic[Title/Abstract] OR diastolic[Title/Abstract]  #6 #4 OR #5  #7 "Diet"[Mesh] OR "Dietary Supplements"[Mesh]  #8 diet*[Title/Abstract] OR supplement*[Title/Abstract] OR oral[Title/Abstract] OR intake[Title/Abstract]  #9 #7 OR #8  #10 #3 AND #6 AND #9  #11 "Clinical Trial" [Publication Type] OR "Cohort Studies"[Mesh]  #12 "clinical trial" OR "randomized controlled trial" OR "randomised controlled trial" OR "longitudinal" OR "cohort"  #13 #11 OR #12  #14 #10 AND #13  Limit: English language |
| EMBASE | #1 exp magnesium/  #2 magnesium.ab,ti  #3 #1 or #2  #4 exp elevated blood pressure/  #5 "blood pressure".ab,ti or hypertens*.ab,ti or systolic.ab,ti or diastolic.ab,ti  #6 #4 or #5  #7 exp diet/ or exp diet therapy/ or exp dietary supplement/  #8 diet.ab,ti or supplement*.ab,ti or oral.ab,ti or intake.ab,ti  #9 #7 or #8  #10 #3 and #6 and #9  #11 exp clinical trial/ or exp cohort analysis/ or exp case control study  #12 ("clinical trial" or "randomized controlled trial" or "randomised controlled trial" or "longitudinal" or "cohort").af  #13 #11 or #12  #14 #10 and #13  Limit: Human and English language |
| Web of Science | #1 Magnesium (Topic)  #2 TS=("blood pressure" or "hypertens*" or "systolic" or "diastolic")  #3 TS=(diet or supplement* or oral or intake)  #4 #1 AND #2 AND #3  #5 ALL=("clinical trial" or "randomized controlled trial" or "randomised controlled trial" or "longitudinal" or "cohort"  #6 #4 AND #5  Limit: English |
| Scopus | #1 TITLE-ABS (magnesium)  #2 TITLE-ABS ("blood pressure" OR "hypertens*" OR "systolic" OR "diastolic" )  #3 TITLE-ABS  ( diet OR supplement* OR oral OR intake )  #4 #1 AND #2 AND #3  #5 ALL ( "clinical trial" OR "randomized controlled trial" OR "randomised controlled trial" OR "cohort" )  #6 #4 AND #5  Limit: Human and English language |
| CINAHL | #1 (MH Magnesium)  #2 TX magnesium  #3 #1 or #2  #4 (MM "Blood Pressure") OR (MH "Hypertension+")  #5 TX "blood pressure" OR TX hypertens* OR TX systolic OR TX diastolic  #6 #4 or #5  #7 (MM "Diet Therapy") OR (MM "Diet")  #8 TX diet OR TX supplement* OR TX oral OR TX intake  #9 #7 or #8  #10 #3 and #6 and #9  #11 (MH "Randomized Controlled Trials+")  #12 TX "clinical trial" OR TX "randomized controlled trial" OR TX "randomised controlled trial" OR TX "longitudinal" OR TX "cohort"  #13 #11 or #12  #14 #10 and #13  Limit: English language |

**Supplementary** **Table S2.** Characteristics of studies included in the meta-analysis

| **Source** | **Study Design; Duration** | **Pre-clinical or Chronic Condition** | **Hypertension Status; use of BP lowering medication** | **Mode of BP Measurement** | **Group** | **Mg Type** | **Elemental Mg Dose (mg)** | **n** | **Age range, mean (yr)** | **Sex** | **Change in SBP (mmHg)** | **Change in DBP (mmHg)** |
| --- | --- | --- | --- | --- | --- | --- | --- | --- | --- | --- | --- | --- |
| Afitska et al.^29^ 2021  Germany | Parallel 12 weeks | Metabolic syndrome, Normomagnesemia | NR  NR | Seated | Treatment | Mg-cit | 400 | 13 | NR, 66 ± 9 | Both | 145.0 ± 10.4 at baseline 120.8 ± 4.6 at endline | 85.4 ± 3.1 at baseline 78.5 ± 2.8 at endline |
|  |  |  |  |  | Placebo |  |  | 11 |  |  | 134.5 ± 6.6 at baseline 133.5 ± 6.6 at endline | 84.0 ± 4.6 at baseline 83.0 ± 3.6 at endline |
| Borello et al.^30^ 1996 Italy | Parallel  12 weeks | Hypertension | Yes No | Supine 24-hr ambulatory | Treatment | MgO | 241 | 42 | 44-58, NR | Both | 155.0 ± 13.0 at baseline 148.5 ± 7.1 at endline | 93 ± 4.0 at baseline 87.5 ± 6.3 at endline |
|  |  |  |  |  | Placebo |  |  | 21 |  |  | 156.0 ± 11.0 at baseline 155.2 ± 8.2 at endline | 92.5± 4.3 at baseline 93.2± 4.5 at endline |
| Cappuccio et al.^31^ 1985  England | Crossover 4 weeks | Hypertension | Yes No | Supine standing | Treatment | MgAsp HCl | 365 | 17 | 33-66,  52 | Both | Supine: 154.0 ± 17.8 at baseline Supine: 154.0 ± 14.0 at endline  Standing: 156.0 ± 15.7 at baseline Standing: 157.0 ± 17.7 at endline | Supine: 100 ± 6.2 at baseline Supine: 98.0 ± 9.1 at endline  Standing: 106.0 ± 7.8 at baseline Standing: 105.0 ± 7.8 at endline |
|  |  |  |  |  | Placebo |  |  | 17 |  |  | Supine: 154.0 ± 12.8 at baseline Standing: 156.0 ± 15.7 at baseline Supine: 151.0 ± 17.3 at endline Standing: 154.0 ± 18.1 at endline | Supine: 100 ± 6.2 at baseline Standing: 106.0 ± 7.3 at baseline Supine: 97.0 ± 8.2 at endline Standing: 106.0 ± 7.4 at endline |
| Cosaro et al.^32^ 2014 Italy | Crossover 8 weeks | Normomagnesemia, family history of metabolic syndrome or T2DM | No No | Seated | Treatment | Mg pidolate | 368 | 16 | 23-33, NR | Male | 126.0 ± 13.1 at baseline 123.7 ± 13.2 at endline | 71.4 ± 5.9 at baseline 71.4 ± 4.5 at endline |
|  |  |  |  |  | Placebo |  |  | 16 |  |  | 122.7 ± 6.5 at baseline 117.2 ± 7.5 at endline | 72.1 ± 5.0 at baseline 69.7 ± 4.1 at endline |
| Cunha et al.^33^ 2017 Brazil | Parallel 24 weeks | Hypertension, with diuretic treatment | Yes Yes | Seated 24-hr ambulatory | Treatment | Mg Chelate | 240 | 17 | 40-65,  54 ± 7 | Female | 144.0 ± 17.0 at baseline 134.0 ± 14.0 at endline | 88.0 ± 9.0 at baseline 81.0 ± 8.0 at endline |
|  |  |  |  |  | Placebo |  |  | 18 |  |  | 143.0 ± 16.0 at baseline 142.0 ± 18.0 at endline | 86.0 ± 10.0 at baseline 86.0 ± 9.0 at endline |
| de Valk et al.^34^ 1998 Netherlands | Parallel 12 weeks | T2DM | NR  NR | Seated | Treatment | MgAsp HCl | 365 | 25 | NR, 63 ± 8.2 | Both | 162.6 ± 23.3 at baseline 154.9 ± 20.7 at endline | 84.0 ± 11.5 at baseline 83.7 ± 9.2 at endline |
|  |  |  |  |  | Placebo |  |  | 25 |  |  | 157.4 ± 23.6 at baseline 147.0 ± 22.4 at endline | 83.0 ± 14.2 at baseline 82.2 ± 16.8 at endline |
| Dykner & Wester^35^ 1983  Sweden | Parallel 24 weeks | Hypertension or congestive heart failure, long term diuretic treatment (>1yr) | NR Yes | Supine Standing | Treatment | MgAsp HCl | 365 | 20 | NR, 62.2 ± 4.2 | Both | Supine: 152.0 ± 20.0 at baseline Supine: 140.0 ± 15.0 at endline  Standing: 145.0 ± 17.0 at baseline Standing: 139.0 ± 18.0 at endline | Supine: 93.0 ± 11.0 at baseline  Supine: 85.0 ± 7.0 at endline Standing: 93.0 ± 13.0 at baseline  Standing: 87.0 ± 10.0 at endline |
|  |  |  |  |  | Placebo |  |  | 19 |  |  | Supine: 154.0 ± 26.0 at baseline  Supine: 154.0 ± 28.0 at endline Standing: 152.0 ± 27.0 at baseline  Standing: 154.0 ± 27.0 at endline | Supine: 90.0 ± 11.0 at baseline  Supine: 86.0 ± 13.0 at endline Standing: 91.0 ± 10.0 at baseline  Standing: 89.0 ± 12.0 at endline |
| Ferrara et al.^36^ 1992 Italy | Parallel 24 weeks | Within 20% of ideal body weight, hypertension | Yes No | Standing Supine | Treatment | Mg pidolate | 341 | 7 | 35-60, NR | Both | Supine: 156.0 ± 12.0 at baseline  Supine: 149.0 ± 8.0 at endline Standing: 155.0 ± 10.0 at baseline  Standing: 155.0 ± 11.0 at endline | Supine: 97.0 ± 4.0 at baseline  Supine: 90.0 ± 3.0 at endline Standing: 102.0 ± 5.0 at baseline  Standing: 96.0 ± 5.0 at endline |
|  |  |  |  |  | Placebo |  |  | 7 |  |  | Supine: 158.0 ± 13.0 at baseline  Supine: 141.0 ± 8.0 at endline Standing: 151.0 ± 6.0 at baseline Standing: 140.0 ± 9.0 at endline | Supine: 93.0 ± 3.0 at baseline  Supine: 89.0 ± 3.0 at endline Standing: 100.0 ± 2.0 at baseline  Standing: 91.0 ± 5.0 at endline |
| Guerrero-Romero et al.^37^ 2004 Mexico | Parallel 12 weeks | Hypomagnesemia, insulin resistance | No No | Seated | Treatment | MgCl | 300 | 32 | NR, 43 ± 7.9 | NR | 110.0 ± 8.4 at baseline 108.0 ± 8.1 at endline | 73.0 ± 7.5 at baseline 72.3 ± 7.4 at endline |
|  |  |  |  |  | Placebo |  |  | 31 |  |  | 111.0 ± 12.0 at baseline 110.0 ± 11.0 at endline | 73.0 ± 9.0 at baseline 72.4 ± 8.9 at endline |
| Guerrero-Romero & Rodriguez-Moran^38^ 2009  Mexico | Parallel 16 weeks | Diabetes, hypomagnesemia on captopril | Yes Yes | Seated | Treatment | MgCl | 450 | 40 | 40-75, NR | Both | 161.1 ± 26.0 at baseline 140.7 ± 11.9 at endline | 88.4 ± 14.5 at baseline 79.7 ± 7.1 at endline |
|  |  |  |  |  | Placebo |  |  | 39 |  |  | 154.5 ± 21.1 at baseline 149.8 ± 20.6 at endline | 84.9 ± 12.4 at baseline 83.8 ± 9.7 at endline |
| Guerrero-Romero & Rodriguez-Moran^39^ 2011 Mexico | Parallel 12 weeks | Hypomagnesemia | No No | Seated | Treatment | MgCl | 637 | 49 | 40-60, NR | Both | 117.2 ± 12.0 at baseline 108.9 ± 9.5 at endline | 73.8 ± 9.4 at baseline 70.3 ± 9.1 at endline |
|  |  |  |  |  | Placebo |  |  | 48 |  |  | 115.9 ± 17.2 at baseline 113.1 ± 10.1 at endline | 73.8 ± 9.6 at baseline 74.1 ± 8.9 at endline |
| Hasan et al.^40^ 2023 Iraq | Parallel 8 weeks | Metabolic syndrome | Yes NR | Seated | Treatment | Mg l-lactate | 168 | 30 | 30-60 | Female | 131.5 at baseline 121.8 ± 9.3 at endline | 79.2 at baseline 75.8 ± 6.0 at endline |
|  |  |  |  |  | Placebo |  |  | 17 |  |  | 131.5 at baseline 130.3 ± 9.5 at endline | 79.2 at baseline 79.1 ± 5.7 at endline |
| Hatzistavri et al.^41^ 2009 Greece | Parallel 12 weeks | SBP 140-159 mmHg and/or DBP 90-99 mmHg) | Yes No | 24-hr ambulatory | Treatment | Mg pidolate | 102 | 24 | NR, 45.3 ± 10.1 | Both | 146.7 ± 4.1 at baseline 141.1 ± 4.1 at endline | 91.5 ± 2.6 at baseline 88.7 ± 2.9 at endline |
|  |  |  |  |  | Placebo |  |  | 24 | NR, 46.9 ± 8.7 |  | 144.7 ± 4.6 at baseline 143.4 ± 5.4 at endline | 89.6 ± 3.9 at baseline 89.5 ± 3.8 at endline |
| Henderson, Schierup & Schodt^42^ 1986 Denmark | Parallel 24 weeks | Hypertension | Yes Yes | Seated | Treatment | MgO | 301 | 20 | NR, 62 | NR | 154.0 ± 19.0 at baseline 150.0 ± 20.0 at endline | 87.0 ± 6.0 at baseline 88.0 ± 7.0 at endline |
|  |  |  |  |  | Placebo |  |  | 20 |  |  | 157.0 ± 24.0 at baseline 154.0 ± 22.0 at endline | 93.0 ± 8.0 at baseline 92.0 ± 2.0 at endline |
| Itoh, Kawasaki & Nakamura^43^ 1997 Japan | Parallel 4 weeks | Hypertension and/or mild hyperlipidaemia | NR  NR | Seated | Treatment | Mg(OH)_2_ | Male: 548 Female: 411 | 23 | NR, 64 | Both | 130 ± 14.0 at baseline 125.0 ± 13.0 at endline | 77.0 ± 9.0 at baseline 75.0 ± 10.0 at endline |
|  |  |  |  |  | Placebo |  |  | 10 | NR, 66 |  | 121.0 ± 15.0 at baseline 122.0 ± 16.0 at endline | 74.0 ± 12.0 at baseline 73.0 ± 10.0 at endline |
| Joris et al.^44^ 2016 Netherlands | Parallel 24 weeks | Healthy, post-menopausal women | NR  NR | Seated 24-hr ambulatory | Treatment | Mg-cit | 350 | 26 | 45-70, 62 ± 6 | Both | 130 ± 15.0 at baseline 126.0 ± 14.0 at endline | 82.0 ± 8.0 at baseline 79.0 ± 8.0 at endline |
|  |  |  |  |  | Placebo |  |  | 25 |  |  | 126.0 ± 14.0 at baseline 123.0 ± 12.0 at endline | 81.0 ± 7.0 at baseline 79.0 ± 7.0 at endline |
| Lee et al.^45^ 2009 Korea | Parallel 12 weeks | Normomagnesemia, BMI ≥ 23kg/m^2^ | NR  NR | Seated | Treatment | MgO | 300 | 75 | 30-60, NR | Both | 124.7 ± 12.3 at baseline 119.2 ± 11.7 at endline | 83.5 ± 9.7 at baseline 80.6 ± 9.3 at endline |
|  |  |  |  |  | Placebo |  |  | 80 |  |  | 126.7 ± 13.5 at baseline 122.8 ± 13.9 at endline | 83.3 ± 9.6 at baseline 82.7 ± 10.3 at endline |
| Lind et al.^46^ 1991 Sweden | Parallel 24 weeks | Hypertension | Yes No | Supine Standing | Treatment | Mg lactate & Mg-cit | 380 | 49 | NR, 60 ± 9.4 | Both | Supine: 151.0 ± 14.0 at baseline Supine: 152.0 ± 14.0 at endline  Standing: 149.0 ± 16.0 at baseline Standing: 151.0 ± 16.0 at endline | Supine: 91.8 ± 6.2 at baseline Supine: 89.1 ± 7.4 at endline Standing: 96.3 ± 6.8 at baseline Standing: 97.0 ± 7.2 at endline |
|  |  |  |  |  | Placebo |  |  | 22 | NR, 62 ± 7.8 |  | Supine: 148.0 ± 12.0 at baseline Supine: 146.0 ± 11.0 at endline  Standing: 146.0 ± 15.0 at baseline  Standing: 145.0 ± 15.0 at endline | Supine: 93.1 ± 5.1 at baseline Supine: 88.9 ± 7.3 at endline  Standing: 98.3 ± 6.4 at baseline  Standing: 97.1 ± 8.0 at endline |
| Lutsey et al.^47^ 2018 USA | Parallel 12 weeks | Healthy | NR  NR | Seated | Treatment | MgO | 242 | 29 | NR, 61.3 ± 5.3 | Both | 119.4 ± 14.0 at baseline 118.0 ± 14.0 at endline | 72.0 ± 9.0 at baseline 71.0 ± 8.8 at endline |
|  |  |  |  |  | Placebo |  |  | 30 | NR, 61.6 ± 5.2 |  | 119.0 ± 18.0 at baseline 115.0 ± 14.0 at endline | 71.0 ± 10.0 at baseline 70.8 ± 8.7 at endline |
| Mooren et al.^48^ 2011 Germany | Parallel 24 weeks | BMI ≥ 25kg/m^2^, decreased insulin sensitivity | NR  NR | NR | Treatment | MgAsp HCl | 365 | 25 | 30-70, NR | Both | 137.7 ± 14.9 at baseline 131.4 ± 16.4 at endline | 85.3 ± 9.4 at baseline 81.6 ± 9.8 at endline |
|  |  |  |  |  | Placebo |  |  | 22 |  |  | 134.8 ± 15.0 at baseline 133.1 ± 21.9 at endline | 82.5 ± 9.6 at baseline 83.2 ± 12.1 at endline |
| Paolisso et al.^49^ 1992 Italy | Parallel 8 weeks | Hypertension with long term (>1yr) hydrochlorothiazide treatment | Yes Yes | Supine | Treatment | Mg pidolate | 384 | 9 | NR, 64 ± 3 | Both | 173.0 ± 27.0 at baseline 159.0 ± 12.0 at endline | 96.0 ± 12.0 at baseline 89.0 ± 15.0 at endline |
|  |  |  |  |  | Placebo |  |  | 9 |  |  | 173.0 ± 27.0 at baseline 171.0 ± 24.0 at endline | 96.0 ± 12.0 at baseline 95.0 ± 9.0 at endline |
| Plum-Wirrell, Stegmayr & Wester^50^ 1994 Sweden | Crossover 8 weeks | Hypertension | Yes No | Supine Standing | Treatment | MgAsp HCl | 365 | 39 | 20-59, NR | Both | Supine: 149.9 ± 17.7 at baseline  Supine: 147.5 ± 15.3 at endline Standing: 149.0 ± 19.6 at baseline  Standing: 147.2 ± 18.4 at endline | Supine: 95.7 ± 8.0 at baseline  Supine: 95.3 ± 9.2 at endline Standing: 103.9 ± 8.8 at baseline  Standing: 104.5 ± 9.5 at endline |
|  |  |  |  |  | Placebo |  |  | 39 |  |  | Supine: 150.7 ± 14.3 at baseline  Supine: 149.9 ± 19.4 at endline Standing: 151.4 ± 16.3 at baseline  Standing: 147.5 ± 19.0 at endline | Supine: 97.9 ± 7.6 at baseline  Supine: 97.5 ± 9.9 at endline Standing: 106.2 ± 7.8 at baseline  Standing: 105.3 ± 11.3 at endline |
| Rodriguez-Moran & Guerro-Romero^51^ 2014 Mexico | Parallel 16 weeks | Medically obese, BMI 20 -24.9 kg/m^2^, hypomagnesemia | NR  NR | Seated | Treatment | MgCl | 382 | 24 | 20-60, NR | Both | 111.3 ± 14.5 at baseline 109.4 ± 12.4 at endline | 71.5 ± 6.6 at baseline 68.8 ± 7.4 at endline |
|  |  |  |  |  | Placebo |  |  | 23 |  |  | 112.3 ± 17.1 at baseline 116.6 ± 11.5 at endline | 71.4 ± 9.3 at baseline 76.8 ± 7.6 at endline |
| Rodriguez-Moran et al^52^ 2018 Mexico | Parallel 16 weeks | Metabolic syndrome, hypomagnesemia | NR  NR | Seated | Treatment | MgCl | 382 | 100 | 30-60, NR | Both | 119.4 ± 17.2 at baseline 115.5 ± 17.4 at endline | 75.1 ± 10.9 at baseline 72.7 ± 10.2 at endline |
|  |  |  |  |  | Placebo |  |  | 98 |  |  | 117.9 ± 16.0 at baseline 119.7 ± 17.0 at endline | 73.1 ± 9.6 at baseline 76.5 ± 10.0 at endline |
| Rodriguez-Moran & Guerro-Romero^53^ 2003 Mexico | Parallel 16 weeks | T2DM, hypomagnesemia | NR  NR | Seated | Treatment | MgCl | 637 | 32 | NR, 59.7 ± 8.3 | NR | 148.3 ± 32.3 at baseline 140.2 ± 28.1 at endline | 86.3 ± 17.0 at baseline 82.7 ± 16.4 at endline |
|  |  |  |  |  | Placebo |  |  | 31 | NR, 54.1 ± 9.6 |  | 138.1 ± 25.6 at baseline 135.0 ± 19.6 at endline | 80.5 ± 14.6 at baseline 79.1 ± 13.5 at endline |
| Rylander & Arnaud^54^  2004 Sweden | Parallel 4 weeks | DBP > 90 mmHg | Yes NR | Supine | Treatment | MgSO_4_ | 82.3 | 18 | 45-64, NR | Both | 148.3 ± 10.5 at baseline 147.9 ± 11.5 at endline | 90.4 ± 4.2 at baseline 90.9 ± 6.6 at endline |
|  |  |  |  |  | Placebo |  |  | 18 |  |  | 151.9 ± 9.8 at baseline 148.3 ± 12.4 at endline | 90.1 ± 4.4 at baseline 89.8 ± 5.0 at endline |
| Sacks et al^55^ 1998 USA | Parallel 16 weeks |  | No No | 24-hr ambulatory | Treatment | Mg lactate | 336 | 50 | NR, 39 ± 4.4 | Female | 117.0 ± 10.0 at baseline 116.5 at endline | 74.0 ± 7.0 at baseline 73.5 at endline |
|  |  |  |  |  | Placebo |  |  | 103 | NR, 38 ± 4.5 |  | 115.0 ± 8.0 at baseline 115.4 at endline | 73.0 ± 6.0 at baseline 73.3 at endline |
| Saito et al^56^ 1988 Japan | Crossover 4 weeks | Hypertension | Yes Yes | Supine | Treatment | MgO | 600 | 20 | NR, 56.6 ± 2 | Both | 134.2 ± 16.1 at baseline 126.7 ± 11.6 at endline | 80.4 ± 11.2 at baseline 77.4 ± 8.1 |
|  |  |  |  |  | Placebo |  |  | 20 |  |  | 134.2 ± 16.1 at baseline 129.6 ± 14.8 at endline | 80.4 ± 11.2 at baseline 78.2 ± 9.4 at endline |
| Salehidoost et al.^57^ 2022 Iran | Parallel 12 weeks | Pre-diabetes | No No | Seated | Treatment | MgO | 250 | 34 | 18-64, 56.7 ± 5.9 | Both | 120.0 ± 20.1 at baseline 115.7 ± 12.8 at endline | 76.8 ± 13.1 at baseline 73.9 ± 9.2 at endline |
|  |  |  |  |  | Placebo |  |  | 37 | 18-64, 54.8 ± 4.9 |  | 120.5 ± 13.3 at baseline 113.0 ± 24.5 at endline | 79.2 ± 15.7 at baseline 74.1 ± 13.0 at endline |
| Schutten et al^58^ 2022a Netherlands | Parallel 24 weeks | BMI 25-35 kg/m^2^ | NR  NR | Supine | Treatment | Mg-cit | 450 | 46 | 45-75, 63.2 ± 6.8 | Both | 133.0 ± 15.0 at baseline 133.0 ± 16.0 at endline | 79.0 ± 9.0 at baseline 79.0 ± 9.0 at endline |
| Schutten et al^58^ 2022b |  |  |  |  | Treatment | MgO | 450 | 46 |  |  | 127.0 ± 15.0 at baseline 127.0 ± 15.0 at endline | 77.0 ± 9.0 at baseline 76.0 ± 9.0 at endline |
| Schutten et al^58^ 2022c |  |  |  |  | Treatment | MgSO_4_ | 450 | 46 |  |  | 130.0 ± 15.0 at baseline 131.0 ± 16.0 at endline | 79.0 ± 9.0 at baseline 79.0 ± 9.0 at endline |
|  |  |  |  |  | Placebo |  |  | 26 |  |  | 130.0 ± 14.0 at baseline 129.0 ± 13.0 at endline | 80.0 ± 8.0 at baseline 80.0 ± 9.0 at endline |
| Simental-Mendia, Rodriguez-Moran & Guerrero-Romero^59^ 2014 Mexico | Parallel 12 weeks | Pre-diabetes, hypomagnesemia | No No | Seated | Treatment | MgCl | 382 | 29 | 18-65, 39.8 ± 16 | Both | 114.8 ± 31.1 at baseline 117.5 ± 18.6 at endline | 76.9 ± 12.9 at baseline 75.0 ± 14.5 at endline |
|  |  |  |  |  | Placebo |  |  | 28 |  |  | 115.7 ± 21.4 at baseline 123.4 ± 22.2 at endline | 72.3 ± 10.5 at baseline 76.9 ± 10.8 at endline |
| Toprak et al.^60^ 2017 Turkey | Parallel 12 weeks | Pre-diabetes, BMI ≥30 kg/m^2^, mild-to-moderate CKD and hypomagnesemia | NR NR | Seated | Treatment | MgO | 365 | 57 | 20-70, NR | NR | 130.7 ± 14.4 at baseline 125.7 ± 14.6 at endline | 73.4 ± 10.1 at baseline 70.4 ± 8.6 at endline |
|  |  |  |  |  | Placebo |  |  | 60 |  |  | 131.5 ± 17.7 at baseline 131.5 ±6.8 at endline | 74.1±12.6 at baseline 74.1 ± 11.2 at endline |
| Walker et al.^61^ 2002 United Kingdom | Parallel 10 weeks | Hypertension | Yes No | Seated | Treatment | Mg amino acid chelate | 600 | 9 | NR, 53.2 ± 3.8 | Both | 150.0 ± 10.5 at baseline 141.0 ± 9.3 at endline | 95.7 ± 6.6 at baseline 93.6 ± 7.2 at endline |
|  |  |  |  |  | Placebo |  |  | 10 | NR, 49.4 ± 4.1 |  | 154.5 ± 13.0 at baseline 139.6 ± 13.9 at endline | 100.0 ± 14.2 at baseline 92.2 ± 11.7 at endline |
| Wary et al.^62^ 1999 Belgium | Parallel 4 weeks | Healthy | No No | Supine Standing | Treatment | Mg lactate | 288 | 15 | NR, 23.7 ± 4.5 | Male | Supine: 113.0 ± 12.0 at baseline  Supine: 111.0 ± 8.0 at endline Standing: 127.0 ± 11.0 at baseline Standing: 121.0 ± 12.0 at endline | Supine: 69.0 ± 8.0 at baseline  Supine: 68.0 ± 7.0 at endline Standing: 76.0 ± 7.7 at baseline Standing: 76.0 ± 5.0 at endline |
|  |  |  |  |  | Placebo |  |  | 15 | NR, 23.7 ± 4.5 |  | Supine: 116.0 ± 11.0 at baseline  Supine: 115.0 ± 7.0 at endline Standing: 126.0 ± 11.0 at baseline Standing: 127.0 ± 7.0 at endline | Supine: 68.0 ± 5.0 at baseline  Supine: 69.0 ± 6.0 at endline Standing: 77.0 ± 5.0 at baseline Standing: 76.0 ± 5.0 at endline |
| Whelton et al.^63^ 1992 USA | Parallel 24 weeks | Healthy | No No | Seated | Treatment | Mg diclycine | 360 | 227 | 35-54, NR | Both | 124.9 ± 8.0 at baseline 122 at endline | 83.8 ± 2.7 at baseline 80.9 at endline |
|  |  |  |  |  | Placebo |  |  | 234 |  |  | 125.4 ± 8.8 at baseline 122.7 at endline | 83.9 ± 2.8 at baseline 80.9 at endline |
| Wirrell, Wester & Stegmayr^64^ 1994a | Crossover 8 weeks | Hypertension | Yes Yes | Supine Standing | Treatment | MgAsp HCl | 365 | 19 | 26-69, NR | Both | Supine: 148.8 ± 14.0 at baseline Supine: 145.0 ± 20.3 at endline  Standing: 143.9 ± 17.6 at baseline  Standing: 140.7 ± 21.4 at endline | Supine: 95.0 ± 6.2 at baseline Standing: 105.5 ± 10.4 at baseline Supine 93.3 ± 10.4 at endline Standing: 101.0 ± 9.9 at endline |
|  |  |  |  |  | Placebo |  |  | 20 |  |  | Supine: 148.8 ± 14.0 at baseline Supine: 152.0 ± 19.3 at endline  Standing: 143.9 ± 17.6 at baseline  Standing: 147.9 ± 25.3 at endline | Supine: 95.0 ± 6.2 at baseline Standing: 105.5 ± 10.4 at baseline Supine: 97.3 ± 8.0 at endline Standing: 104.0 ± 9.1 at endline |
| Wirrell, Wester & Stegmayr^64^ 1994b | Crossover 8 weeks | Hypertension | Yes Yes | Supine Standing | Treatment | MgAsp HCl | 365 | 20 | 26-69, NR | Both | Supine: 147.0 ± 18.7 at baseline Supine: 145.1 ± 16.2 at endline  Standing: 147.0 ± 26.0 at baseline  Standing: 142.4 ± 15.4 at endline | Supine: 95.1 ± 8.1 at baseline Standing: 103.1 ± 7.5 at baseline Supine: 92.8 ± 8.4 at endline Standing: 100.7 ± 9.1 at endline |
|  |  |  |  |  | Placebo |  |  | 19 |  |  | Supine: 147.0 ± 18.0 at baseline Supine: 147.0 ± 18.0 at endline  Standing: 147.0 ± 26.0 at baseline  Standing: 144.3 ± 22.1 at endline | Supine: 95.1 ± 8.1 at baseline Standing: 103.1 ± 7.5 at baseline Supine: 94.3 ± 10.2 at endline Standing: 101.2 ± 11.7 at endline |
| Witteman et al^65^ 1994 Netherlands | Parallel 24 weeks | Hypertension | Yes No | Seated | Treatment | MgAsp HCl | 485 | 47 | 35-77, NR | Female | 146.2 ± 13.6 at baseline 143.8 ± 14.0 at endline | 89.4 ± 6.7 at baseline 86.1 ± 7.0 at endline |
|  |  |  |  |  | Placebo |  |  | 44 |  |  | 146.4 ± 11.2 at baseline 146.6 ± 13.5 at endline | 90.0 ± 7.0 at baseline 90.1 ± 6.9 at endline |
| Zemel et al^66^ 1990 USA | Parallel 12 weeks | Hypertension | Yes No | Supine | Treatment | MgAsp HCl | 486 | 7 | 20-69, NR | Both | 145.0 ± 10.6 at baseline 148.0 ± 15.9 at endline | 90.0 ± 5.3 at baseline 92.0 ± 5.3 |
|  |  |  |  |  | Placebo |  |  | 6 |  |  | 140.0 ± 12.2 at baseline 139.0 ± 12.2 at endline | 89.0 ± 5.9 at baseline 90 ± 9.8 at endline |

Abbreviations: NR, not reported; Mg-cit, magnesium citrate; MgO, magnesium oxide; MgAsp HCl, magnesium aspartate hydrochloride; T2DM, type 2 diabetes; CKD, chronic kidney disease; BMI, body mass index; MgCl, magnesium chloride; Mg(OH)_2_, magnesium hydroxide; MgSO_4_, magnesium sulphate

**Supplementary Table S3.** Risk-of-Bias 2 (RoB2) assessments of studies included in meta-analysis (N=38)

**
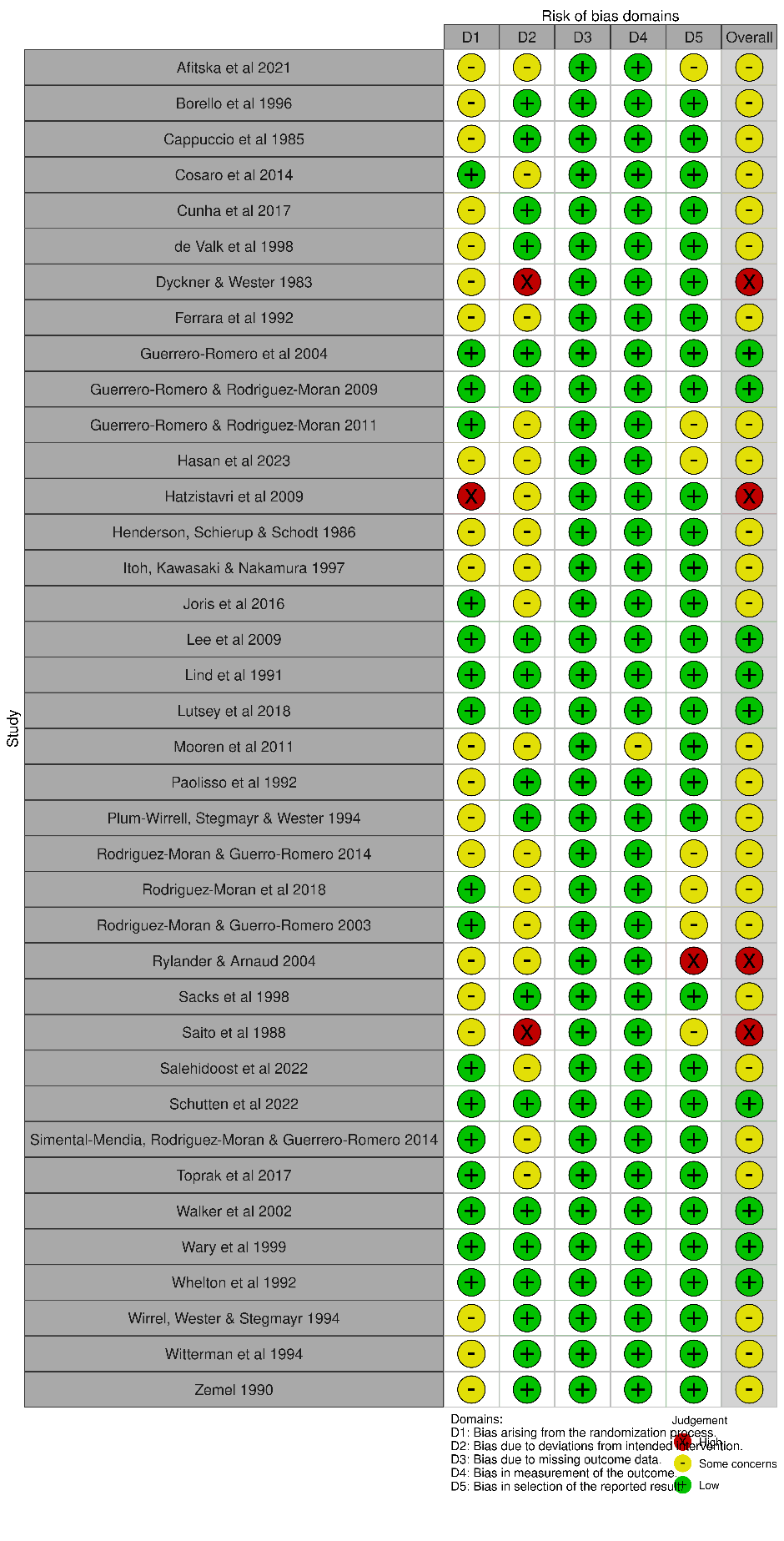
**

| **Domains:** | **Judgement** |
| --- | --- |
| D1: Bias arising from the randomization process | **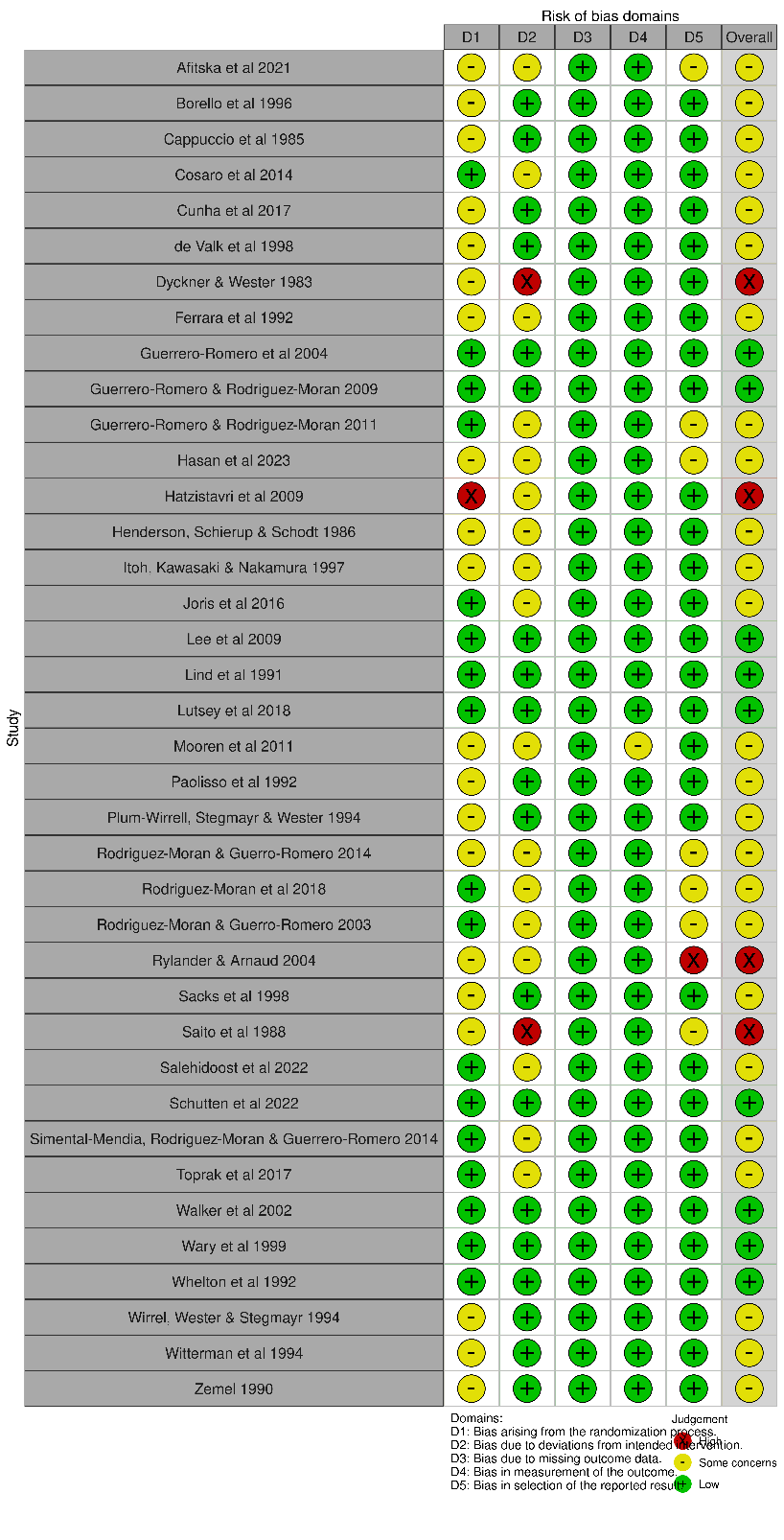**  Low    Some concerns  High |
| D2: Bias due to deviations from intended intervention |  |
| D3: Bias due to missing outcome data |  |
| D4: Bias in measurement of outcome |  |
| D5: Bias in selection of reported results |  |

**Supplementary Table S4**. Results from dose-response meta-analysis of relationship between magnesium intake and changes in SBP and DBP.

| **P values** | **P – linear** | **P – non linear** |
| --- | --- | --- |
| SBP difference – intervention | 0.6703 | 0.5632 |
| SBP difference – control | 0.3797 | 0.2048 |
| DBP difference – intervention | 0.6817 | 0.6123 |
| DBP difference – control | 0.4984 | 0.4106 |

**Supplementary Figure S1.** Funnel plot for change in SBP


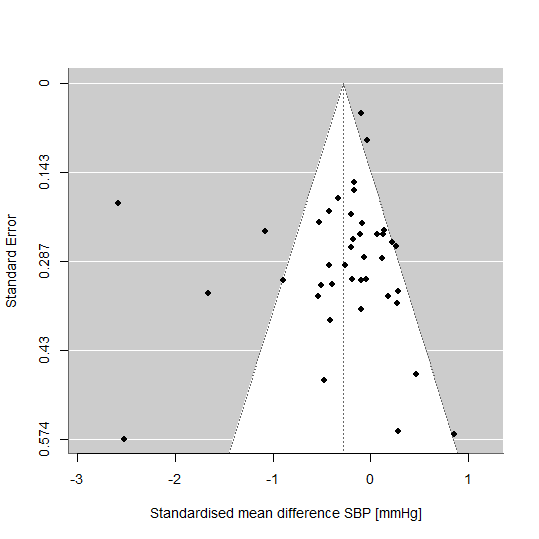


**Supplementary Figure S2.** Funnel plot for change in DBP


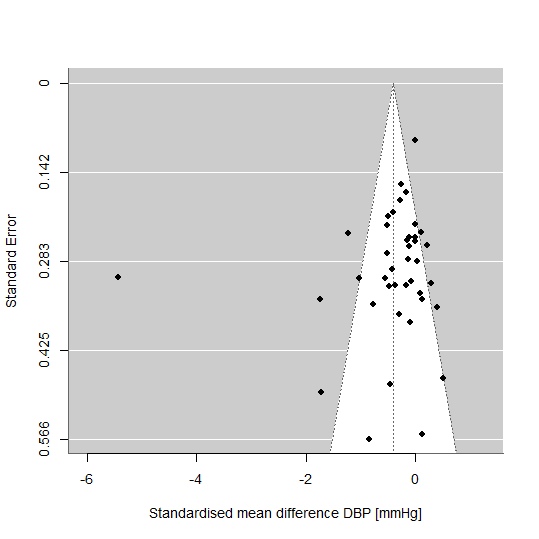


**Supplementary Figure S3.** Forest plot of change in DBP (mmHg) among participants with hypertension (N=18) and normotension (N=8).


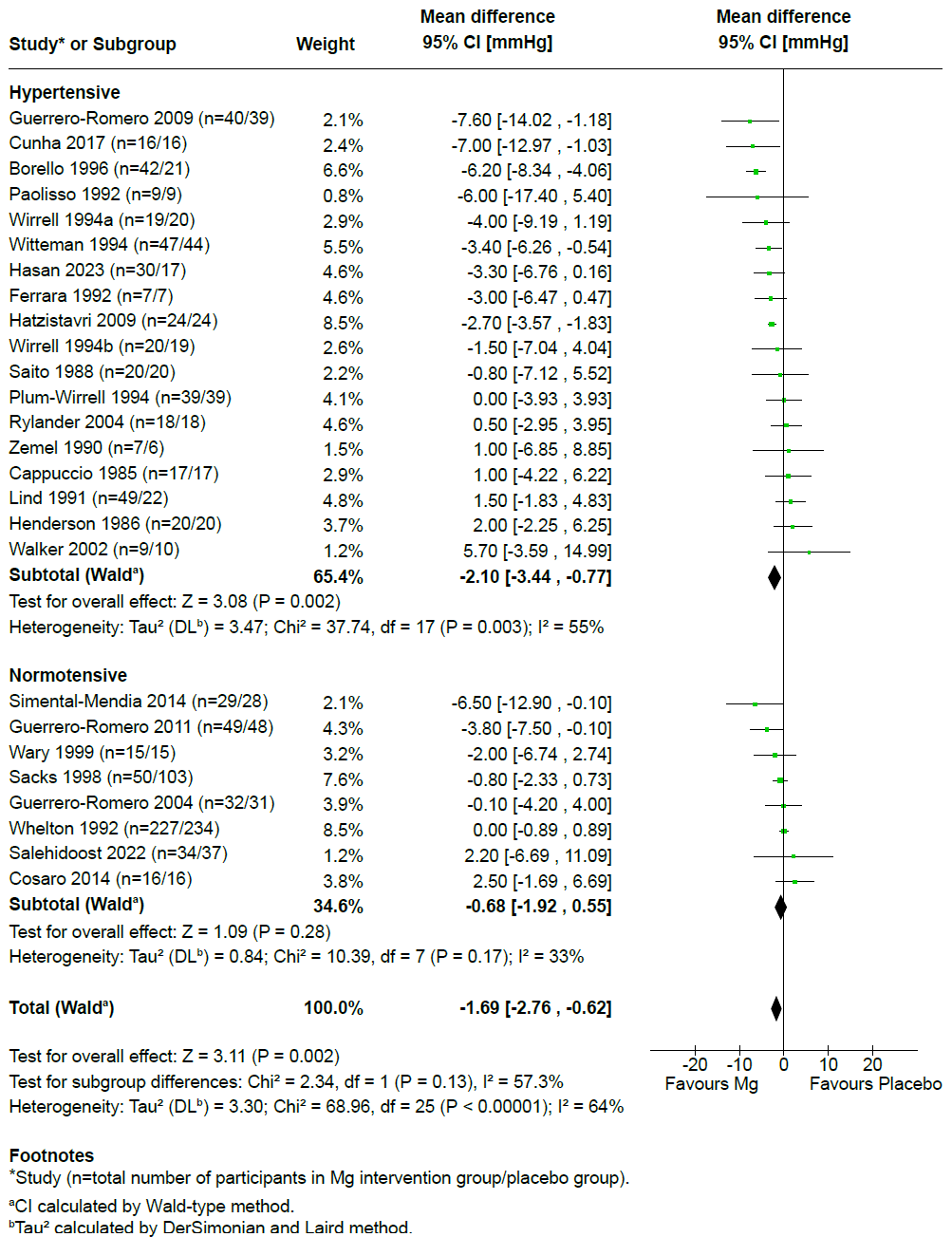


**Supplementary Figure S4.** Forest plot of change in SBP (mmHg) among studies based on hypertension and medication status.


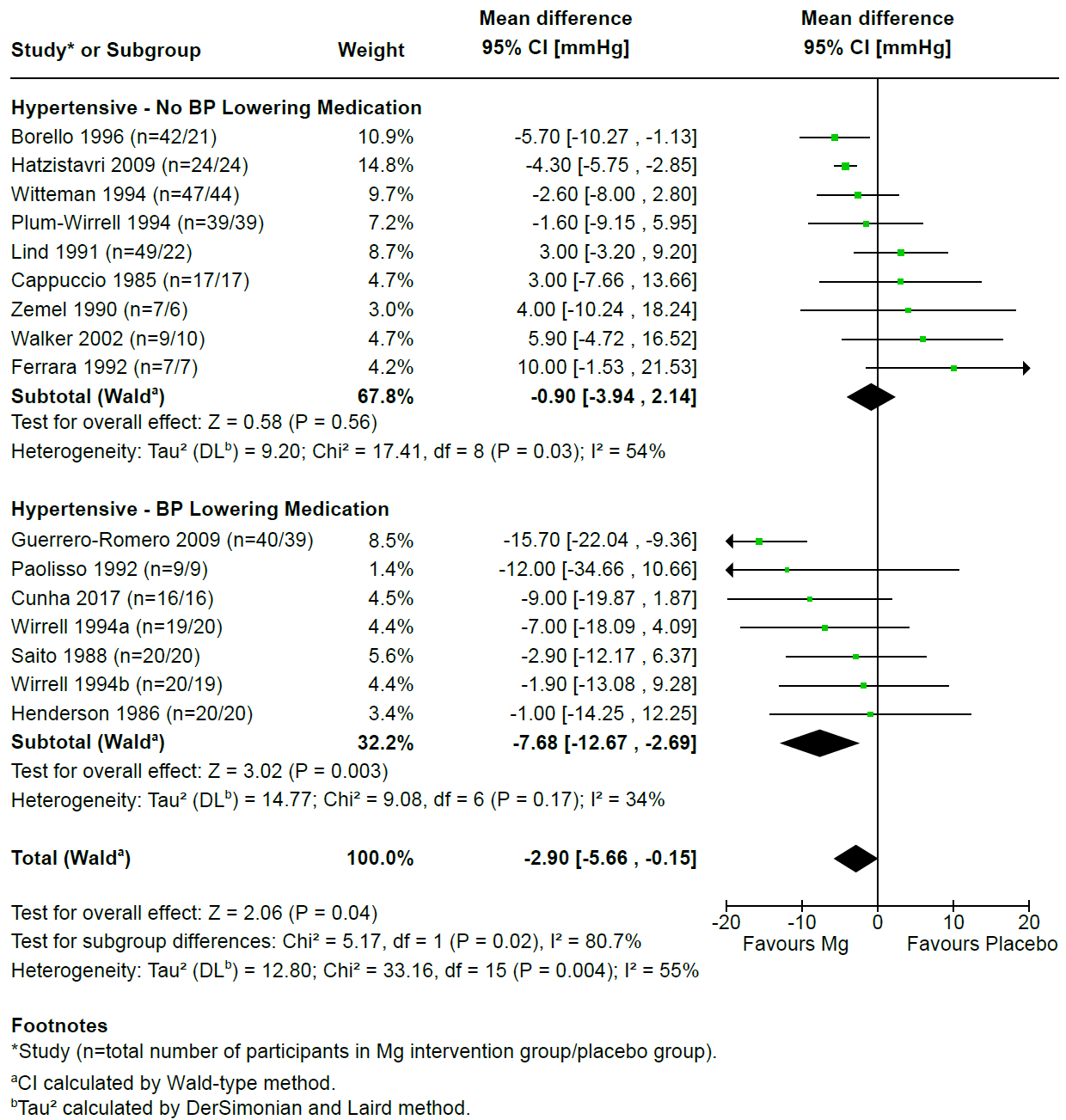


**Supplementary Figure S5.** Forest plot of change in DBP (mmHg) among studies based on hypertension and medication status.


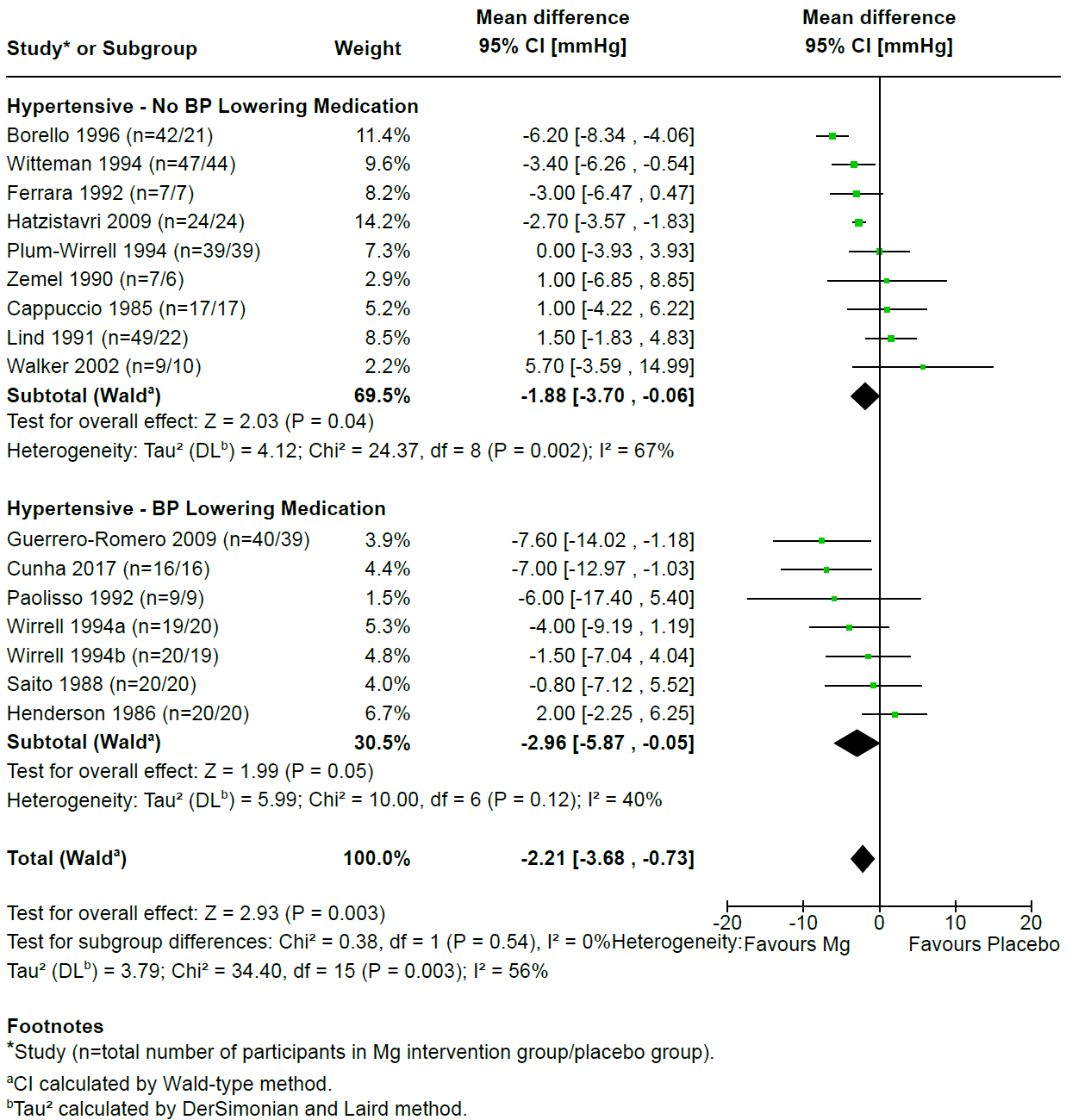


**Figure S6.** Forest plot of change in SBP (mmHg) among participants with hypomagnesemia (N=8) and normomagnesemia (N=3).

**
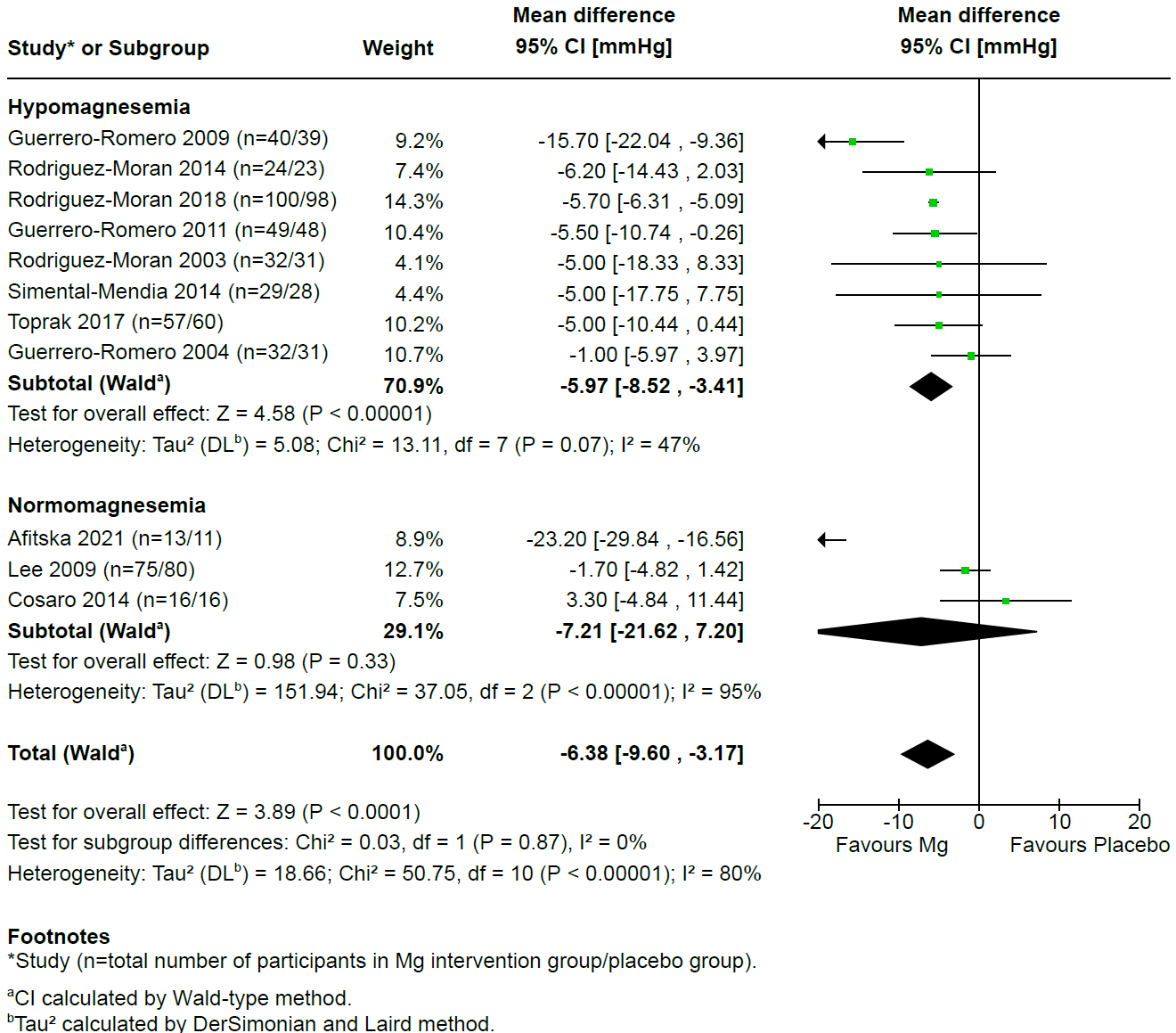
**

**Figure S7.** Forest plot of change in DBP (mmHg) among participants with hypomagnesemia (N=8) and normomagnesemia (N=3).


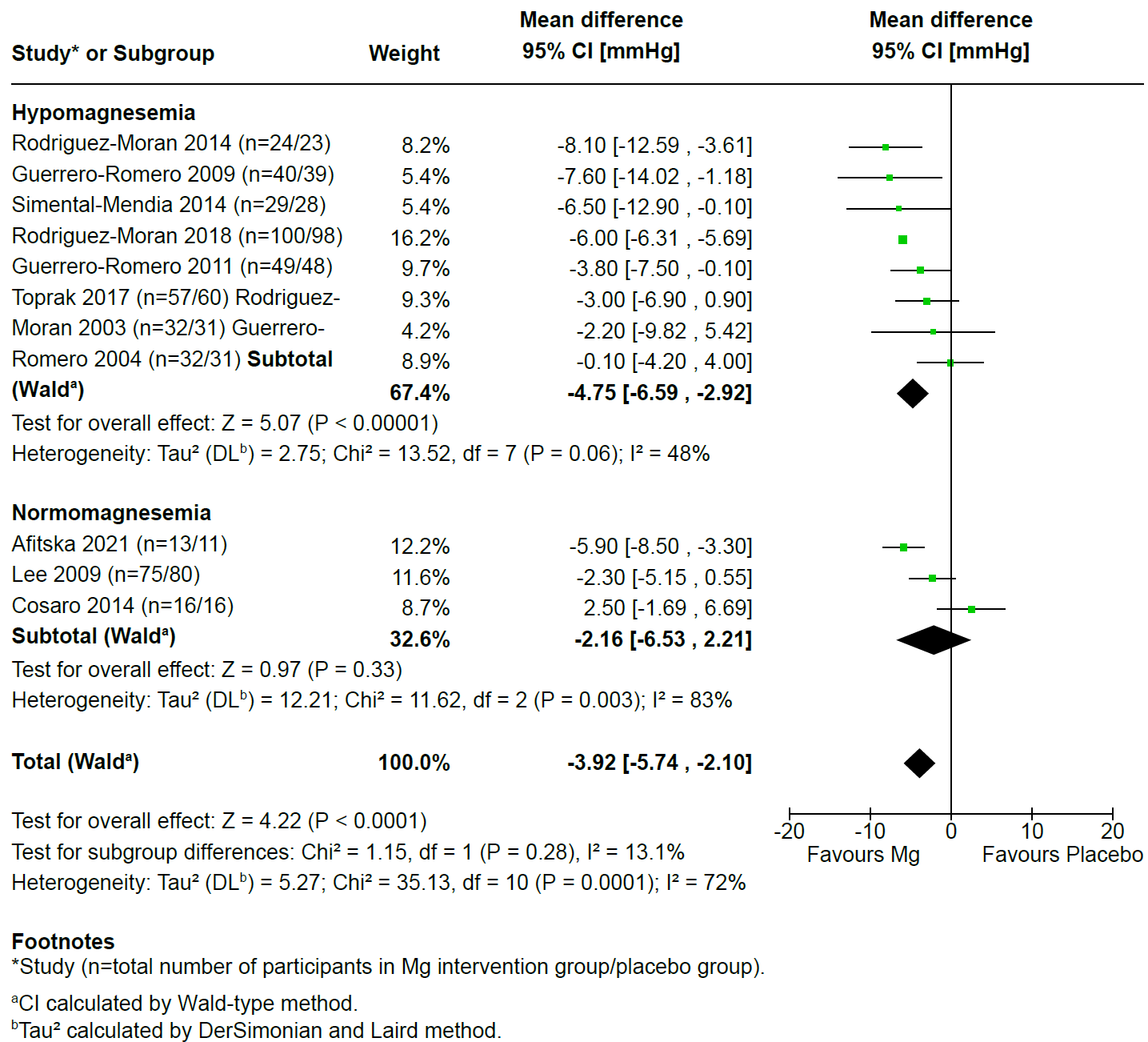


**Supplementary Figure S8**. Forest plot of change in SBP (mmHg) among studies based on body position of blood pressure measurement.


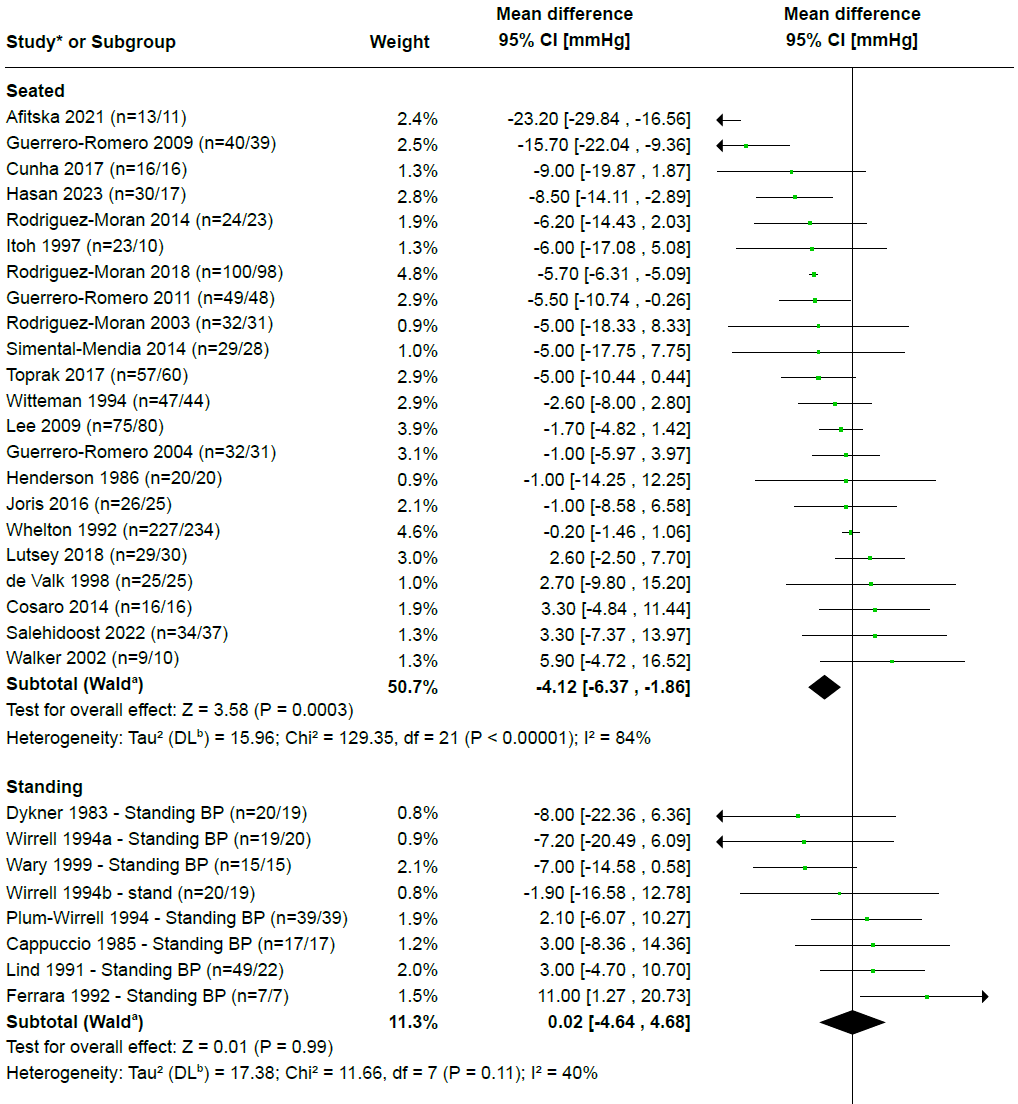


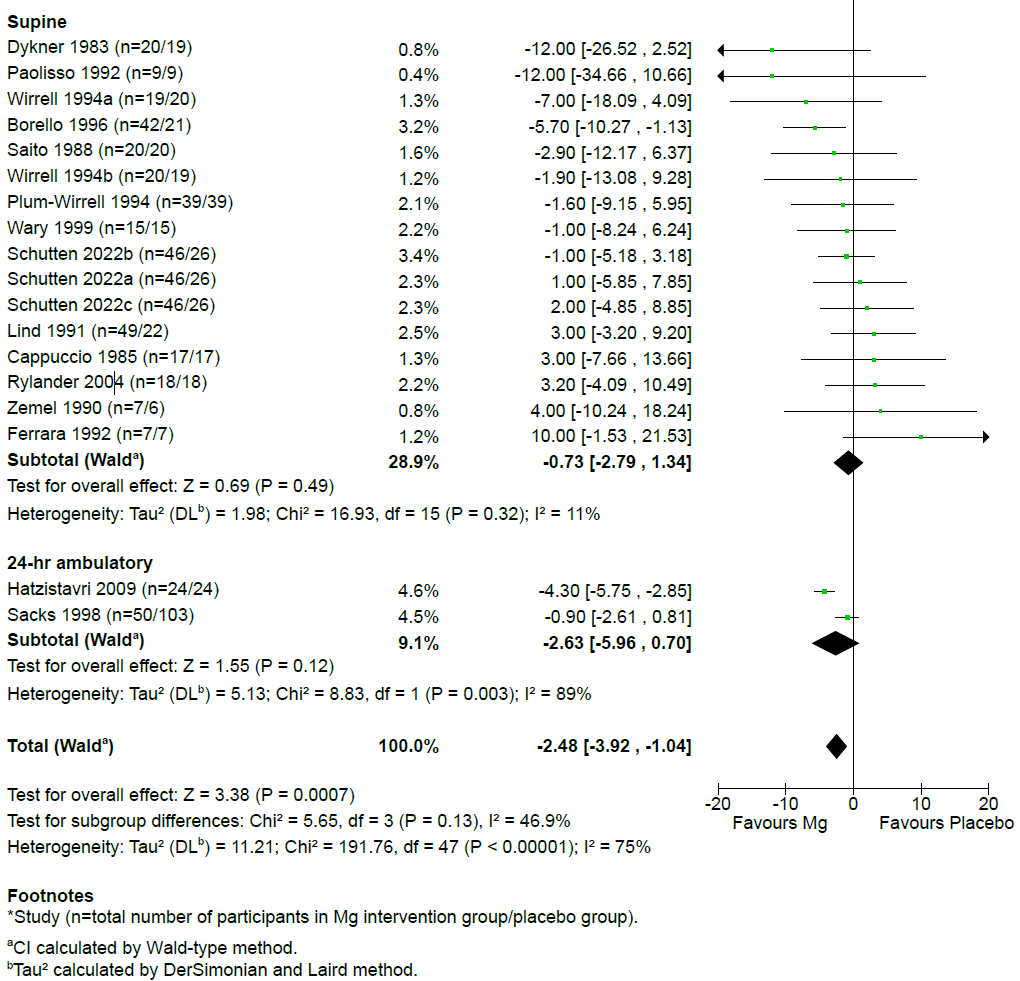


**Supplementary Figure S9**. Forest plot of change in DBP (mmHg) among studies based on body position of blood pressure measurement.


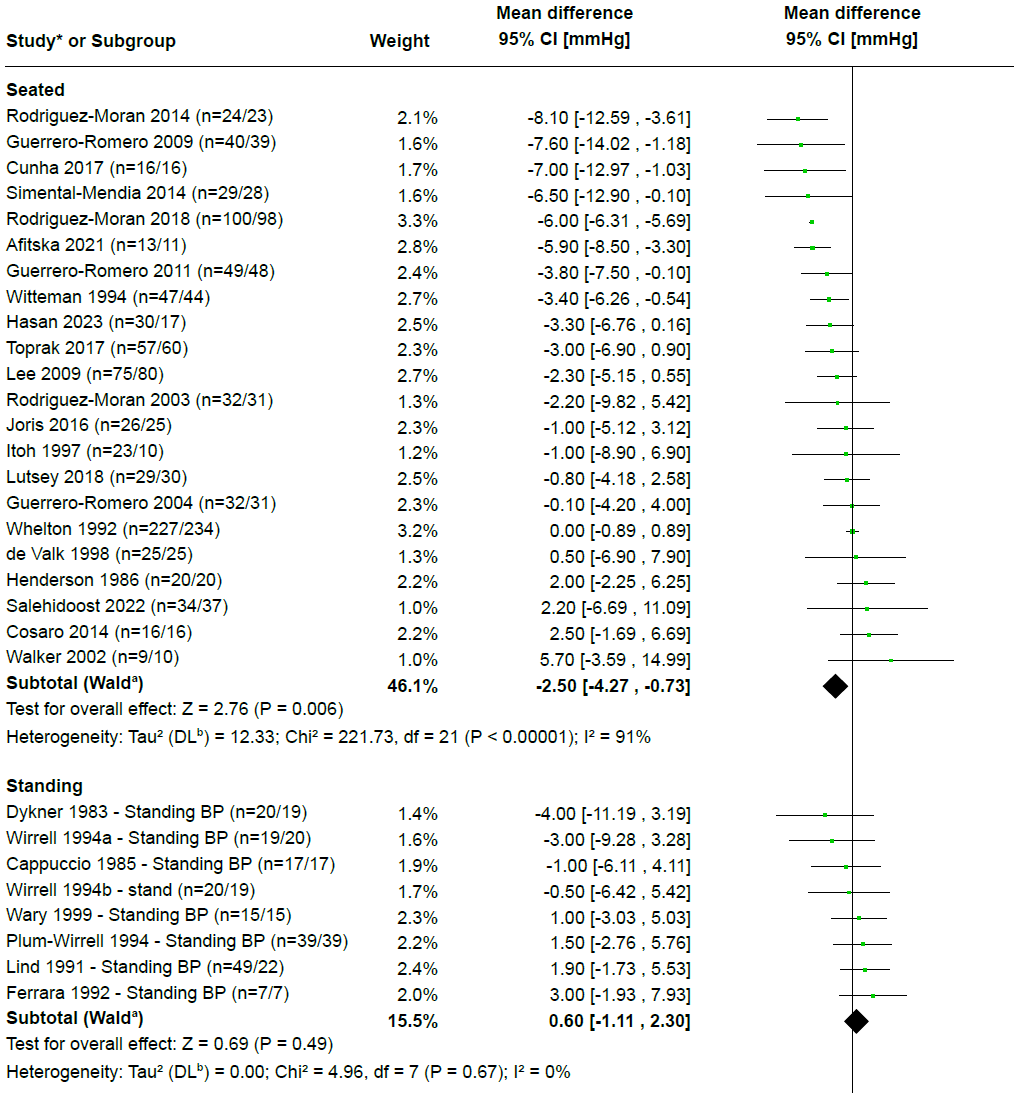


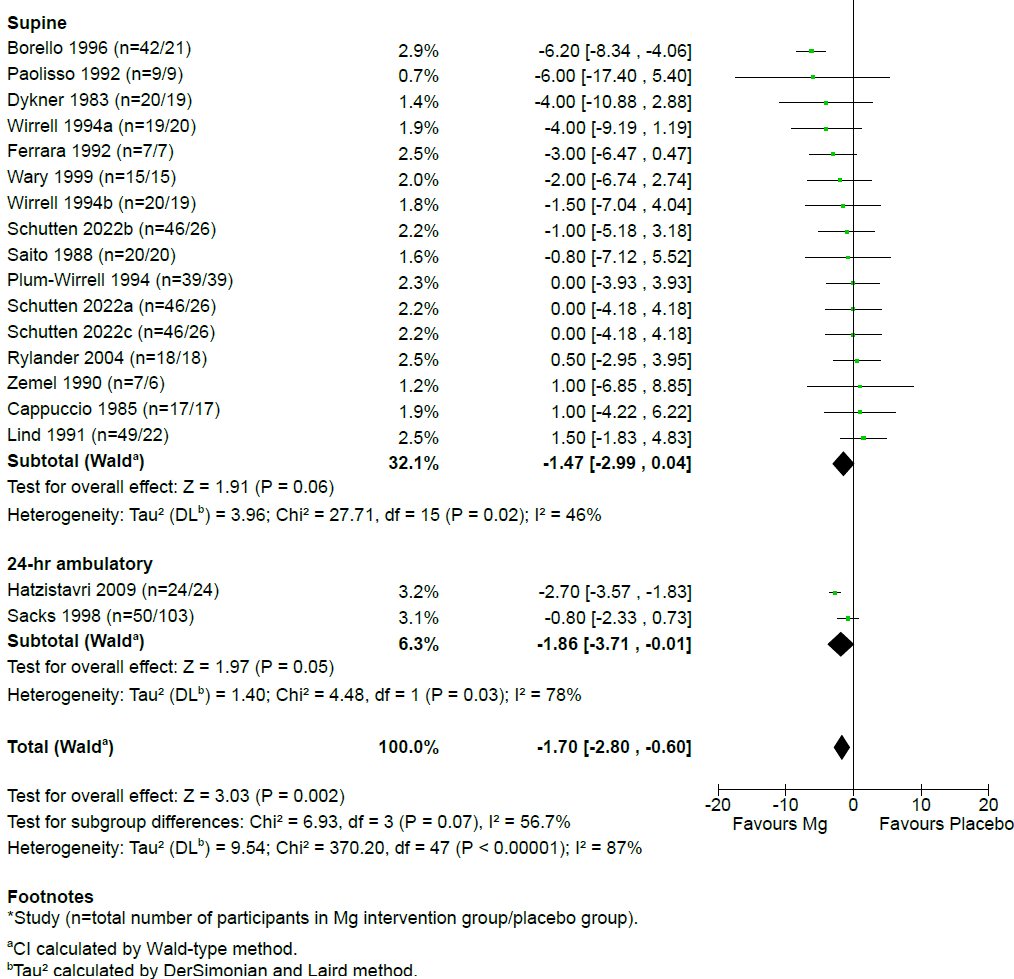


**Supplementary Figure S10**. Forest plot of change in SBP (mmHg) among studies based on study design.


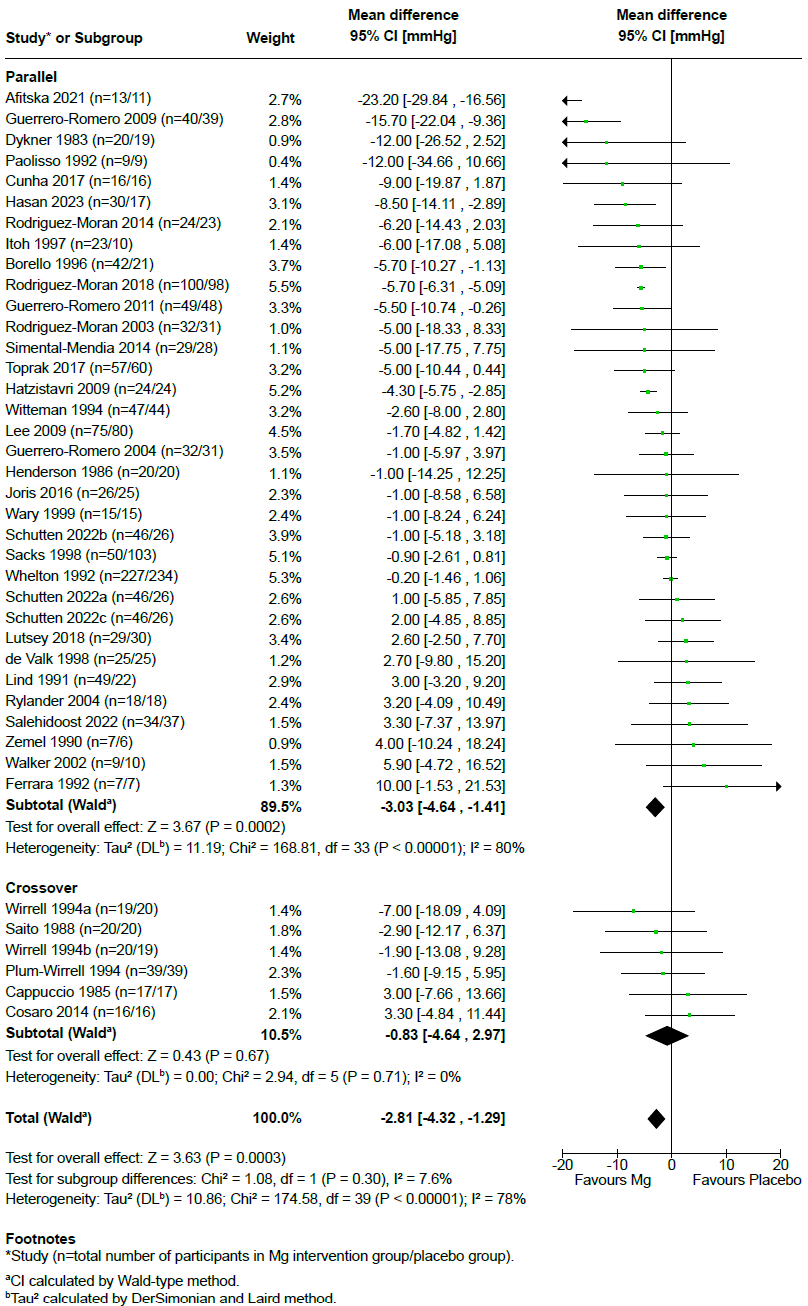


**Supplementary Figure S11**. Forest plot of change in DBP (mmHg) among studies based on study design.


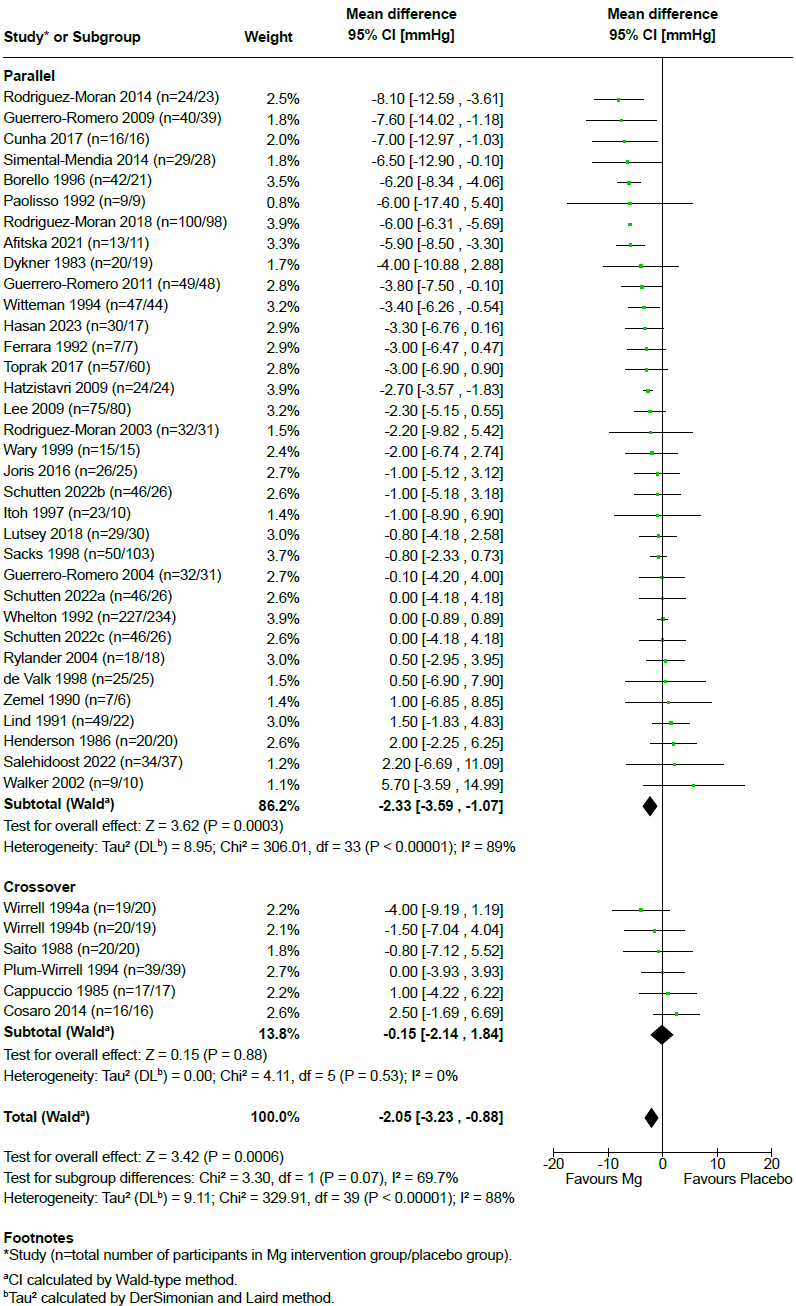


**Supplementary Figure S12**. Forest plot of change in SBP (mmHg) among studies based on magnesium supplement type.


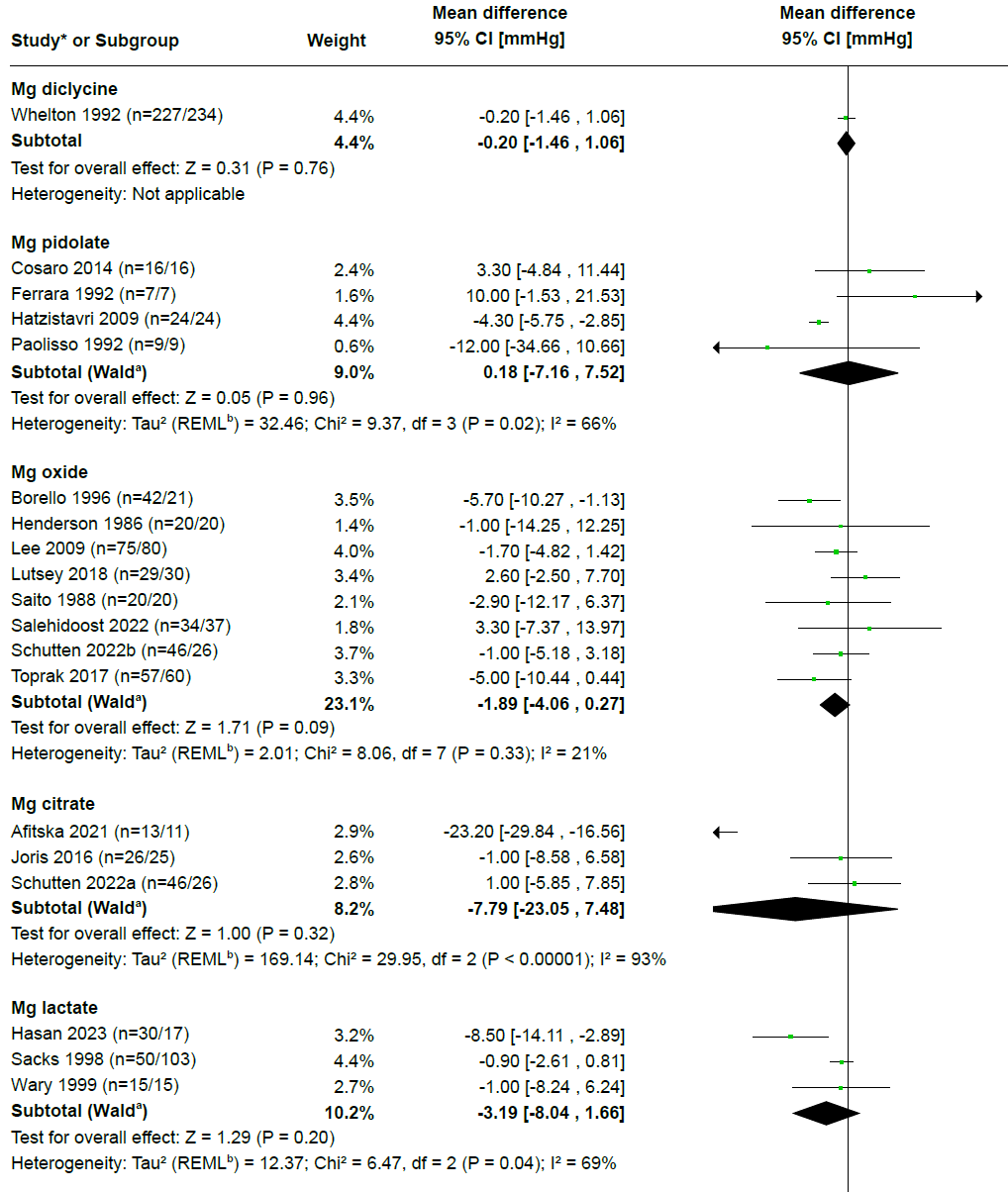


**
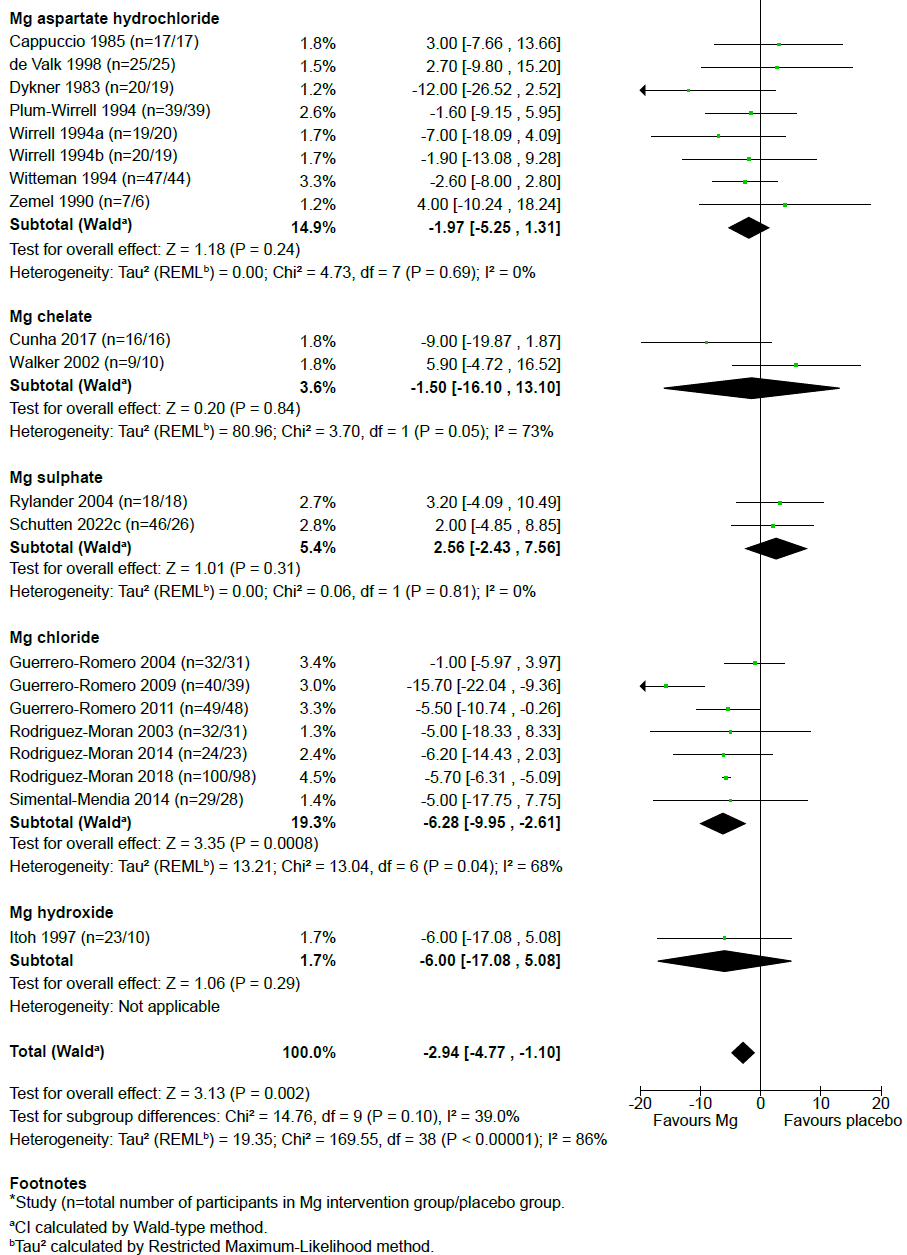
**

**Supplementary Figure S13**. Forest plot of change in DBP (mmHg) among studies based on magnesium supplement type.

**
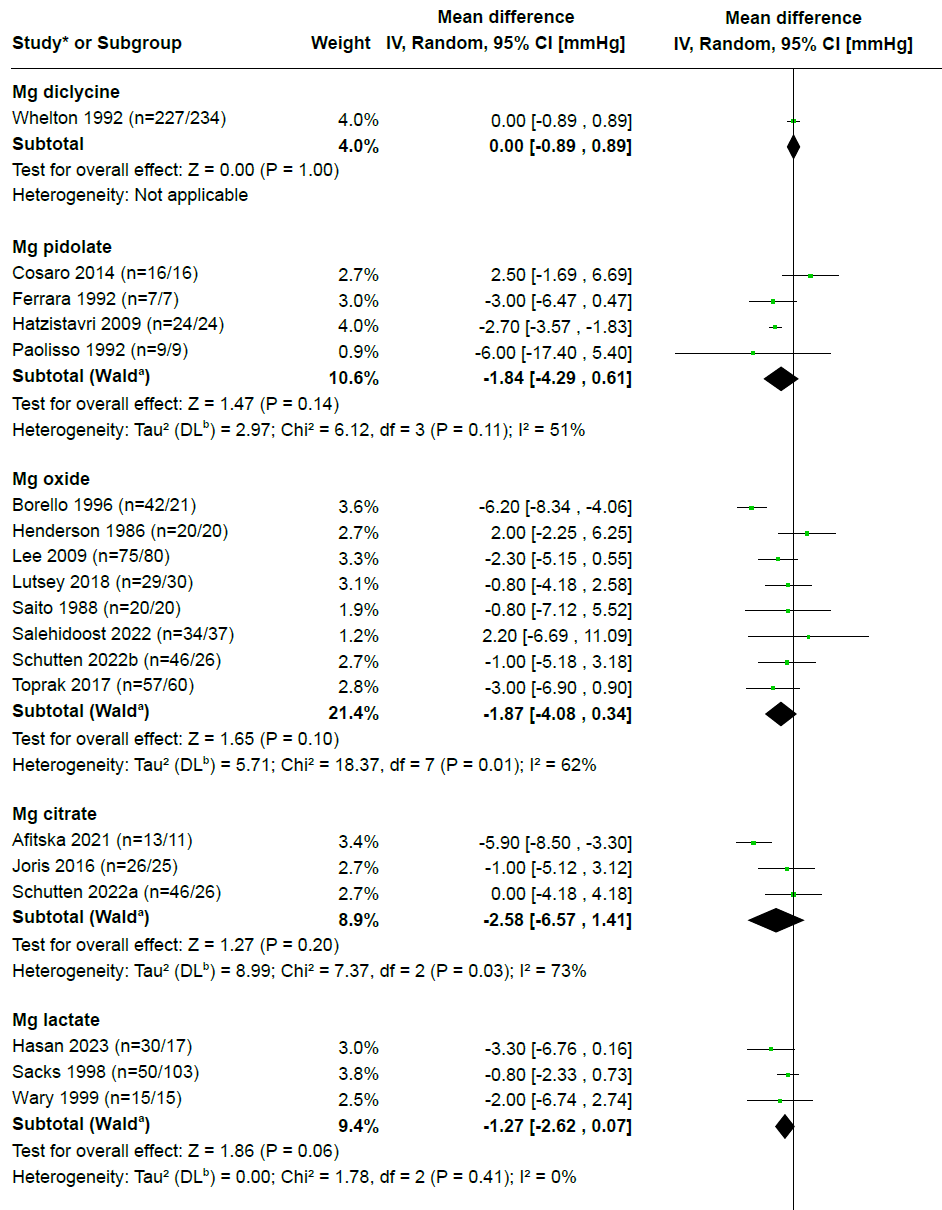
**

**
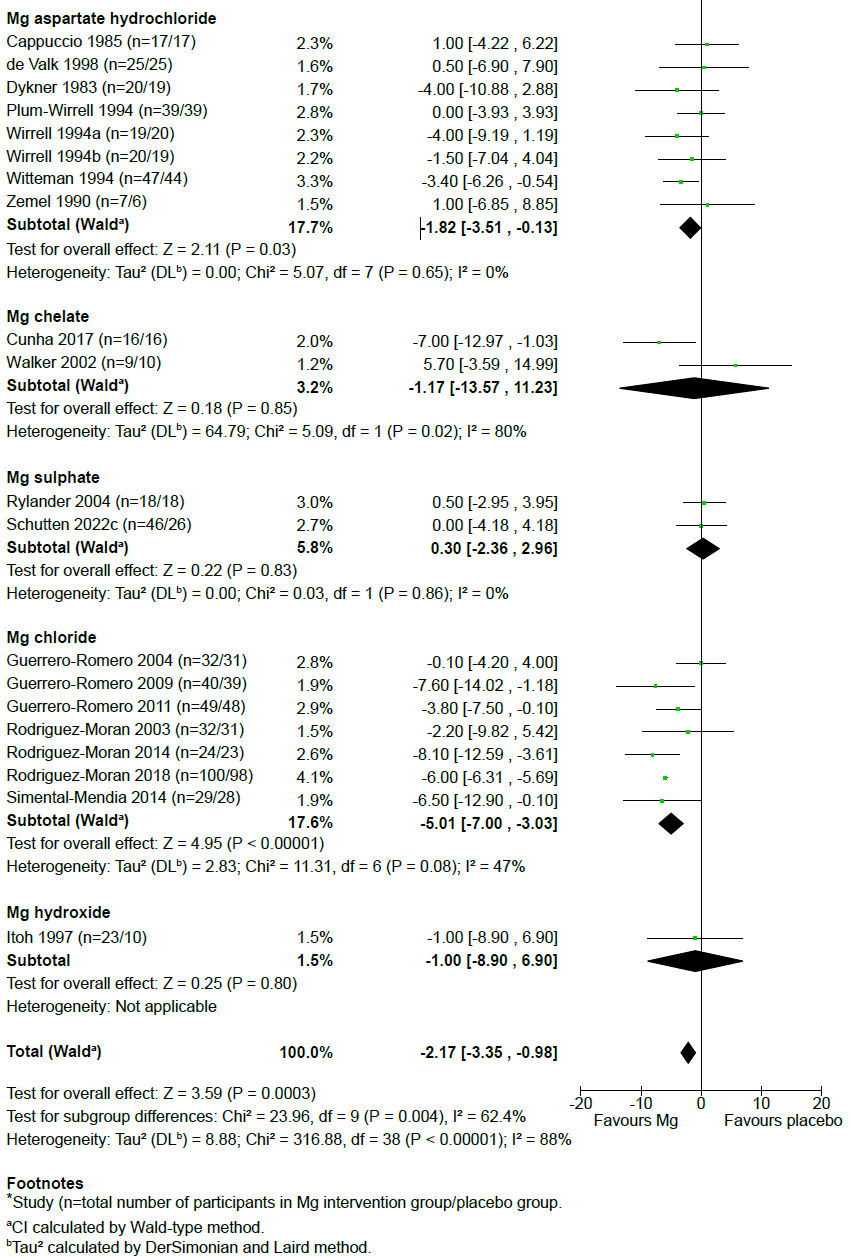
**
